# Insight of vitellogenesis patterns: A comparative analysis of the differences between the primary and secondary vitellogenesis period in the ovary, hepatopancreas, and muscle of mud crab, *scylla paramamosain*

**DOI:** 10.3389/fgene.2022.965070

**Published:** 2022-08-29

**Authors:** Yuanhao Ren, Wei Wang, Zhiqiang Liu, Minghao Luo, Yin Fu, Fengying Zhang, Chunyan Ma, Ming Zhao, Wei Chen, Keji Jiang, Lingbo Ma

**Affiliations:** ^1^ Key Laboratory of East China Fishery Resources Exploitation, Ministry of Agriculture and Rural Affairs, East China Sea Fisheries Research Institute, Chinese Academy of Fishery Science, Shanghai, China; ^2^ College of Fisheries and Life Sciences, Shanghai Ocean University, Shanghai, China

**Keywords:** *Scylla* paramamosain, vitellogenesis patterns, ovarian development, ovarian re-maturation, biochemical components, GSI and HSI

## Abstract

The mud crab, *Scylla paramamosain*, has abundant nutrients in its edible parts, ovary, hepatopancreas, and muscle during the ovarian maturation stage. The ovary of *S. paramamosain* can re-mature after spawning during the secondary ovarian maturation period. We aimed to analyze the characteristics of the first vitellogenesis period (FVP)^1^ and second vitellogenesis period (SVP)^2^ of *S. paramamosain* during ovarian maturation to understand the differences in vitellogenesis patterns between the first and second ovarian maturation periods. Accordingly, the gonadosomatic index (GSI) and hepatopancreatic index (HSI), the external and histological characteristics of the ovary and hepatopancreas, the *Sp-Vg* (vitellogenin, Vg) expression levels in the hepatopancreas and ovary, and the dynamics of the biochemical components in the ovary, hepatopancreas, and muscle were determined. Based on the results, the GSI was significantly positively correlated with HSI during the FVP and significantly negatively correlated with HSI from stage Ⅳ to stage Ⅴ of the SVP. A significant difference was found between the FVP and SVP in the hepatopancreas. Notably, the hepatopancreas displayed a gradual degeneration trend during the SVP. The expression level of *Sp-Vg* was significantly higher in the hepatopancreas than that in the ovary during the FVP and SVP. Seventeen amino acids were detected in the hepatopancreas, ovary, and muscle during the FVP and SVP, with glutamate as the predominant amino acid. During the FVP and SVP, the C16:0 and C18:1n9c were the dominant fatty acids in the hepatopancreas and ovary, the MUFA gradually increased in the ovary and hepatopancreas, and a significant difference was found in the dynamic trend of the HUFA and SFA contents from stage Ⅳ to stage Ⅴ between the FVP and SVP. These findings indicate that the ovary can re-mature after spawning in *S. paramamosain* and can maintain the status of the first ovarian maturation; however, the hepatopancreas gradually degenerate during the SVP.

## Introduction

Mud crabs of the genus, *Scylla* (Portunidae, Decapoda, Crustacea), which include four species, namely *Scylla paramamosain*, *Scylla serrata*, *Scylla olivacea*, and *Scylla tranquebarica*, are commercially valuable crustaceans throughout the Indo-Pacific region and are widely distributed in many tropical and subtropical countries of Asia (Le Vay, 2001; Islam et al., 2010; Zhao et al., 2021). According to the annual China Fishery Statistical Yearbook, approximately 240,000 tons (∼5 million dollars) of mud crabs are obtained annually through aquaculture and fishing (Bureau of Fisheries Ministry of Agriculture PRC, 2021). *Scylla paramamosain* has a high commercial value owing to the abundant nutrients in its edible parts, including the ovary, hepatopancreas, and muscle, during the ovarian maturation period. Therefore, numerous studies have been conducted on the ovarian maturation stages, with vitellogenesis recognized as an essential biological event during the period of ovarian development that has attracted the attention of many researchers (Ma et al., 2012; Huang et al., 2017; Wu et al., 2020; Wan et al., 2021).

The ovarian developmental periods of the *S. paramamosain* are mainly divided into five stages (Stage Ⅰ: undeveloped; Stage Ⅱ: pre-vitellogenesis; Stage Ⅲ: early vitellogenesis; Stage Ⅳ: late vitellogenesis; Stage Ⅴ: mature) based on the characteristics of morphological and histological observations, gonadosomatic index (GSI), and hepatosomatic index (HSI) (Huang et al., 2014; Wu et al., 2020). Of these stages, the vitellogenesis period includes stages Ⅲ, Ⅳ, and Ⅴ (Islam et al., 2010), in which Vg is synthesized by various tissues (hepatopancreas and ovary), undergoes a series of processes and modifications, continuously accumulates in oocytes, and provides nutrients for ovary maturation and embryonic development (Yano & Hoshino, 2006; Subramoniam, 2011). Many species of crab, such as the Chinese mitten crab (*Eriocheir sinensis*) (Liu et al., 2011), swimming crab (*Portunus trituberculatus*) (Wu et al., 2010), and mud crab (*S. paramamosain*) (Yu et al., 2021), can spawn more than once after a single mating, indicating that their ovaries can re-mature during the second ovarian developmental period that equally includes the vitellogenesis period.

Based on previous studies using *E. sinensis* (Ying et al., 2006; Wu et al., 2017), *P. trituberculatus* (Che et al., 2018; Duan et al., 2021), and *S. paramamosain* (Wu et al., 2020), it has been reported that during the ovarian developmental period, many characteristic changes occur, including growth performance, external and histological features, and biochemical composition dynamics in the edible parts. Among these characteristics, the dynamics of the biochemical components such as lipids and amino acids in the ovary, hepatopancreas, and muscles during the ovarian developmental period have been well studied in *S. paramamosain* (Wu et al., 2020). In aquatic crustaceans, lipids, which are critical energy nutrients, mainly include fatty acids, phospholipids, and sterols and are indispensable for many reproduction-related processes, including ovarian development, egg formation, spawning, embryogenesis, and early larval development (Harrison, 1990; Xu et al., 1994). Amino acids serve as an essential nutrient source for ovarian maturation, embryonic development, spawning, and hatching (Peñaflorida, 2004). Previous studies have revealed the biochemical compositions of the edible parts of *S. serrata* (Peñaflorida, 2004; Romano et al., 2014), *S. olivacea* (Taufik et al., 2020), and *S. paramamosain* (Jiang et al., 2014; Tantikitti et al., 2015). Wu et al. (2020) clarified the dynamics of the biochemical composition, including lipids and amino acids, in the ovary, hepatopancreas, and muscle of *S. paramamosain* during the ovarian maturation period. Previous studies mainly concentrated on the first ovarian maturation period before the first spawning; however, owing to the increased economic value and reduced availability of wild crabs, studies on the second ovarian maturation period after spawning has attracted the attention of researchers (Wu et al., 2010). To date, numerous reports have been published on the second ovarian maturation period of many economically important crabs, such as *E. sinensis* (Liu et al., 2011) and *P. trituberculatus* (Yao et al., 2007; Wu et al., 2010), clearly revealing the characteristics of the second ovarian maturation period. However, only a single report was published on the second ovarian maturation period in *S. paramamosain*, which included a comparative analysis of the biochemical composition in the ovary and hepatopancreas between the two ovarian maturation periods during ovarian stage Ⅴ (Yu et al., 2021). Detailed characteristics and differences in the different ovarian stages between the two ovarian maturation periods (the first and second ovarian maturation periods) remain unclear and need to be further studied.

This study aimed to mainly focus on the vitellogenesis periods (ovarian stages Ⅲ, Ⅳ, and Ⅴ) during the two ovarian maturation periods (the first and second ovarian maturation periods) in *S. paramamosain*. The characteristics between the two vitellogenesis periods (the FVP and SVP), which included external and histological characteristics, HSI, GSI, the expression level of *Sp-Vg*, and dynamic changes in biochemical composition, were compared. This study aimed to provide information that will help to better understand the dynamics in *S. paramamosain* during the two vitellogenesis periods. The findings of this study are valuable for subsequent analyses of the vitellogenesis patterns in *S. paramamosain*.

## Materials and methods

### Animals and sampling

We selected 100 individuals of *S. paramamosain* in ovarian stage III of the first ovarian maturation period, sourced from the Research Center of Ninghai, Zhejiang Province, the East China Sea Fisheries Research Institute, Chinese Academy of Fishery Sciences, for the experiment. The animals were reared in rectangular tanks containing seawater at a temperature of 25 ± 2 °C and a salinity of 25 ppt until ovarian maturation. After the first spawning, *S. paramamosain* continued to receive feed until the second ovarian maturation stage. During the 5-month feeding period, all crabs were fed on the razor clam, *Sinonovacula constricta*. During the two vitellogenesis periods, tissue samples were collected from the hepatopancreas (F-Hep-3, F-Hep-4, F-Hep-5, S-Hep-3, S-Hep-4, and S-Hep-5), ovaries (F-OV-3, F-OV-4, F-OV-5, S-OV-3, S-OV-4, and S-OV-5), and muscle (F-Mu-3, F-Mu-4, F-Mu-5, S-Mu-3, S-Mu-4, and S-Mu-5) (see Table 1). Sufficient amounts of each tissue sample were quick-frozen in liquid nitrogen for RNA extraction and biochemical component analysis.

**TABLE 1:** The information of the samples number.

Samples number	Vitellogenesis periods (first or second)	Ovarian stages	Tissues
F-OV-3	First	Ⅲ	ovary
F-OV-4	First	Ⅳ	ovary
F-OV-5	First	Ⅴ	ovary
F-Hep-3	First	Ⅲ	hepatopancreas
F-Hep-4	First	Ⅳ	hepatopancreas
F-Hep-5	First	Ⅴ	hepatopancreas
F-Mu-3	First	Ⅲ	muscle
F-Mu-4	First	Ⅳ	muscle
F-Mu-5	First	Ⅴ	muscle
S-OV-3	Second	Ⅲ	ovary
S-OV-4	Second	Ⅳ	ovary
S-OV-5	Second	Ⅴ	ovary
S-Hep-3	Second	Ⅲ	hepatopancreas
S-Hep-4	Second	Ⅳ	hepatopancreas
S-Hep-5	Second	Ⅴ	hepatopancreas
S-Mu-3	Second	Ⅲ	muscle
S-Mu-4	Second	Ⅳ	muscle
S-Mu-5	Second	Ⅴ	muscle

### Histological analysis and index measurement

The ovarian developmental stages of *S. paramamosain* were determined based on previous studies (Islam et al., 2010; Wu et al., 2020). The wet weights of the ovary, hepatopancreas, and body were measured using a digital microbalance to the nearest 0.001 g. We calculated HSI and GSI by dividing the weights of the ovary and hepatopancreas by the body weight and multiplying by 100 (James et al., 2013; Waiho et al., 2017). For histological analysis, the ovary (F-OV-3, F-OV-4, F-OV-5, S-OV-3, S-OV-4, S-OV-5, F-Hep-3, F-Hep-4, F-Hep-5, S-Hep-3, S-Hep-4, and S-Hep-5) (see Table 1) and hepatopancreas were fixed in Bouin’s solution for 24 h at 4°C and transferred to 70% ethanol. Subsequently, fixed tissues were dehydrated in gradient ethanol concentrations (70–100%), cleared in xylol, embedded in paraffin wax, and cut into 5 μm sections before staining with hematoxylin-eosin (Waiho et al., 2017). Sections were viewed under Pannoramic DESK, P-MIDI, and P250 (3D HISTICH, Magyarország), and the diameters of the oocytes were measured using the CaseViewer software.

### RNA extraction and analysis of the *Sp-Vg* expression level

Total RNA was extracted from the ovary and hepatopancreas using TRIzol Reagent (Invitrogen) and quantified using an ND-2000 NanoDrop UV spectrophotometer (NanoDrop Technologies). Residual DNA in the samples was removed using RNase-free DNase Ⅰ (Sangon Biotech, Shanghai). RNA (2 μg) was subsequently used to synthesize cDNA using the M-MuLV First Strand cDNA Synthesis Kit (Sangon Biotech, Shanghai) following the manufacturer’s protocol.

The expression levels of the *Sp-Vg* transcript during the two vitellogenesis periods were determined using quantitative real-time PCR on the QuantStudio™ 7 Flex System (Applied Biosystems), according to the manufacturer’s instructions for the Trans Strat Tip Green qPCR Super Mix kit (TransGen Biotech, Beijing). The primer sequences of *Sp-Vg*-F, *Sp-Vg*-R, *Sp-18S*-F, and *Sp-18S*-R were listed in Table 2; *Sp-18S* was used as an internal control (Jia et al., 2013; Zhao et al., 2015). All samples were analyzed three times by qPCR using the following cycling conditions: 94°C for 30 s, 40 cycles of 94°C for 5 s, 60°C for 30 s; followed by a melting curve at 94°C for 15 s, 60°C for 1 min, and 94°C for 15 s.

**TABLE 2:** The primer sequences for RT-qPCR used in this study.

Primers	Sequence
*Sp-Vg*-F	5′-CGC​AAC​CGC​CAC​TGA​AGA​T-3′
*Sp-Vg*-R	5′-CCA​CCA​TGC​TGC​TCA​CGA​CT-3′
*Sp-18S*-F	5′-GGG​GTT​TGC​AAT​TGT​CTC​CC-3′
*Sp-18S*-R	5′-GGT​GTG​TAC​AAA​GGG​CAG​GG-3′

### Biochemical components analysis

Three groups were established at each stage of ovarian development. Five crabs were retrieved from each group during the two vitellogenesis periods, and a total of 90 crabs were used to analyze biochemical components. In the present study, the biochemical component data of the ovary, hepatopancreas, and muscle during the two vitellogenesis periods mainly included proximate composition, fatty acid content, and amino acid content, which were detected by Standard Testing Group Co., Ltd., (Qingdao, China) using the methods reported in previous studies (Ying et al., 2006; Wang et al., 2021).

### Statistical analysis

All qPCR data were calculated using the relative standard curve method. Statistical analysis was performed, and the differences in the HSI, GSI, contents of different biochemical components, and the *Sp-Vg* expression levels between the two vitellogenesis periods were analyzed using IBM SPSS software (version 26.0). One-way analysis of variance and Student’s t-test were used to determine statistical significance. Pearson’s correlation test was conducted between the GSI and HSI during the two vitellogenesis periods using IBM SPSS software (version 26.0). *p*-values less than 0.05 were considered significantly different.

## Results

### External and histological characteristics of the ovary and hepatopancreas in the two vitellogenesis periods

**FIGURE 1:**
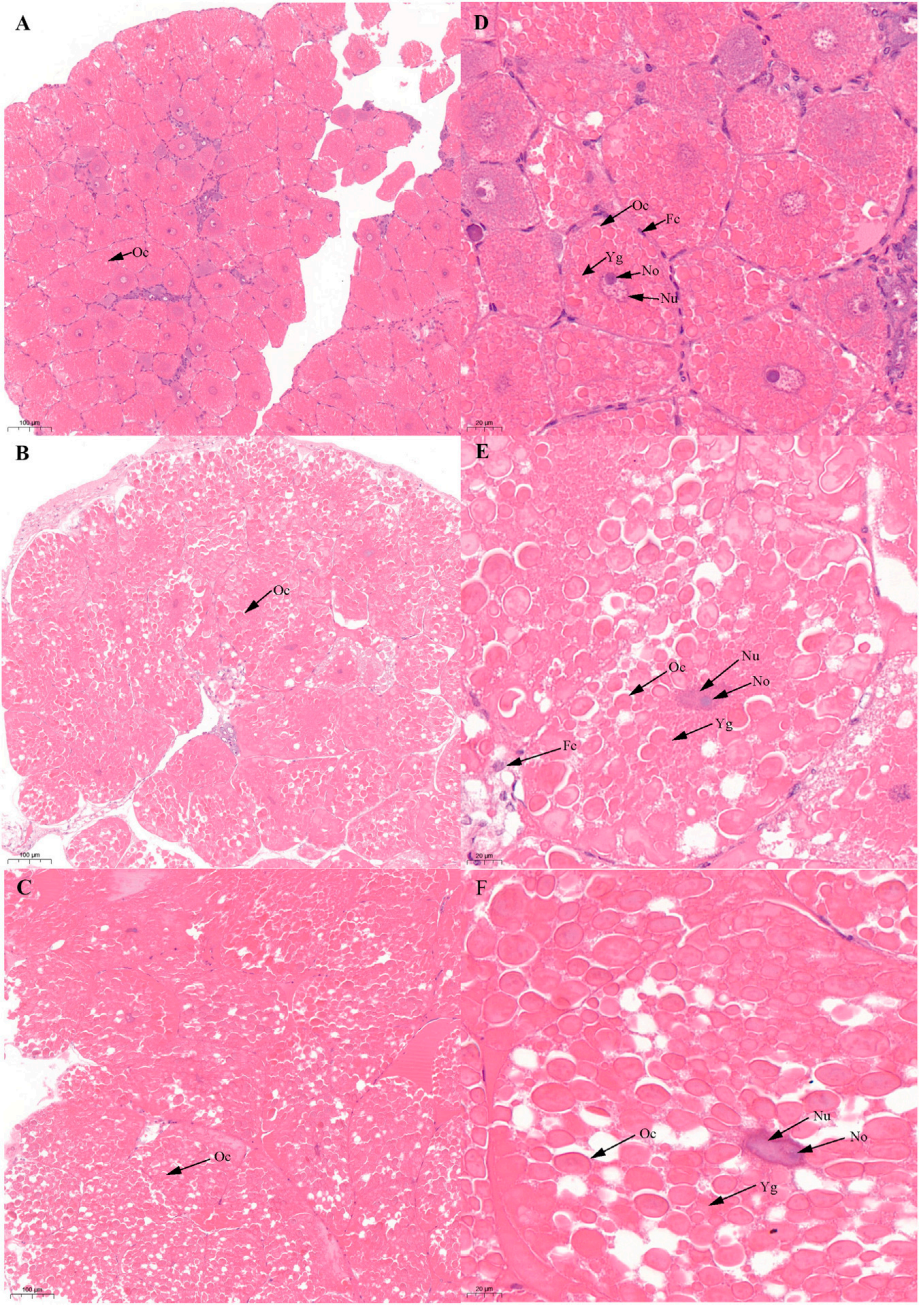
Histological characteristics in the ovary of *Scylla paramamosain* during the FVP. **(A,D)**: ovarian stage Ⅲ (F-OV-3); **(B,E)**: ovarian stage Ⅳ (F-OV-4); **(C,F)**: ovarian stage Ⅴ (F-OV-5). Oc, Oocyte; Fc, Follicular cells; Nu, Nucleus; No, Nucleolus; Yg, Yolk granules.

**FIGURE 2:**
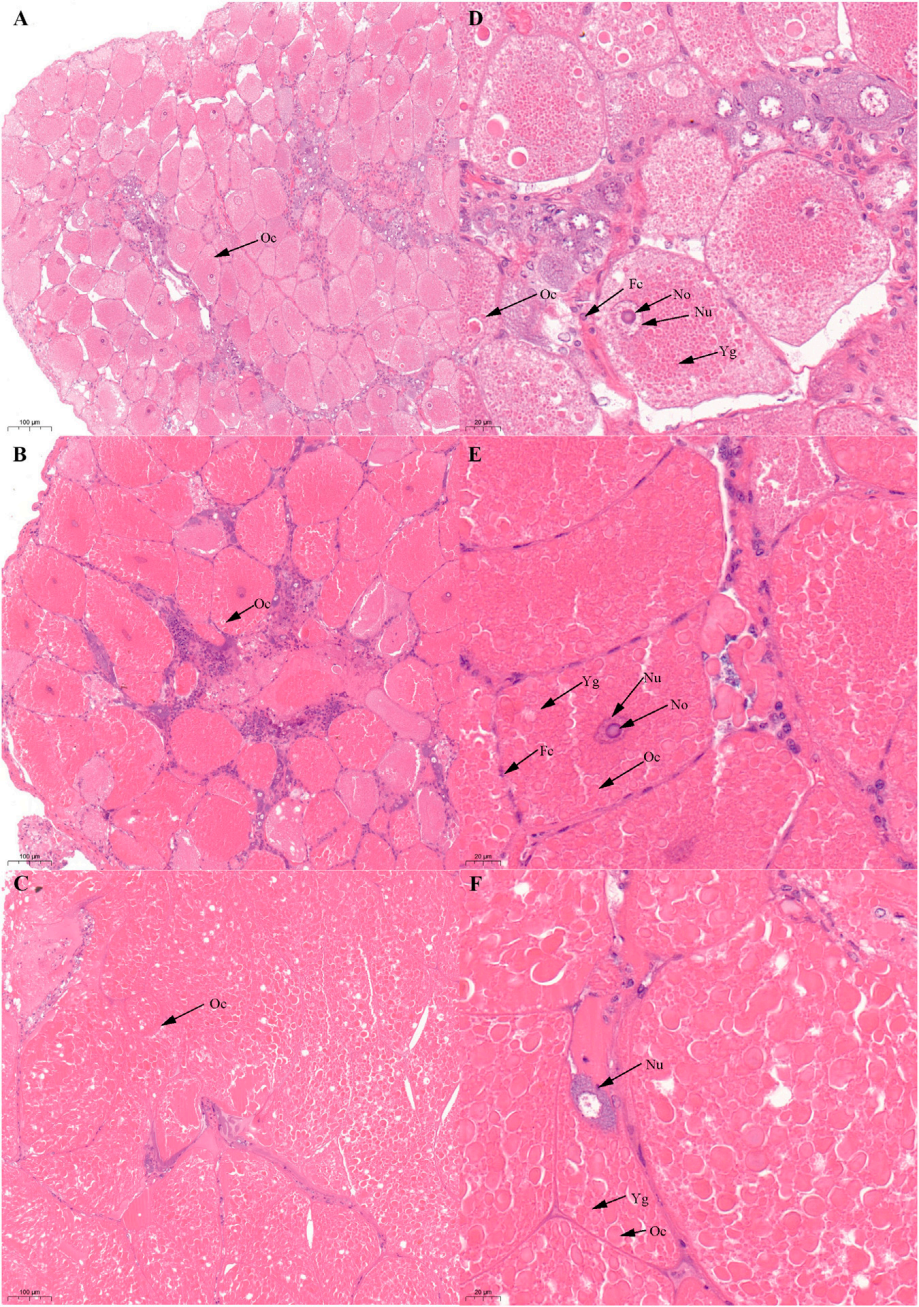
Histological characteristics in the ovary of *Scylla paramamosain* during the SVP. **(A,D)**: ovarian stage Ⅲ (S-OV-3); **(B,E)**: ovarian stage Ⅳ (S-OV-4); **(C,F)**: ovarian stage Ⅴ (S-OV-5). Oc, Oocyte; Fc, Follicular cells; Nu, Nucleus; No, Nucleolus; Yg, Yolk granules.

**FIGURE 3:**
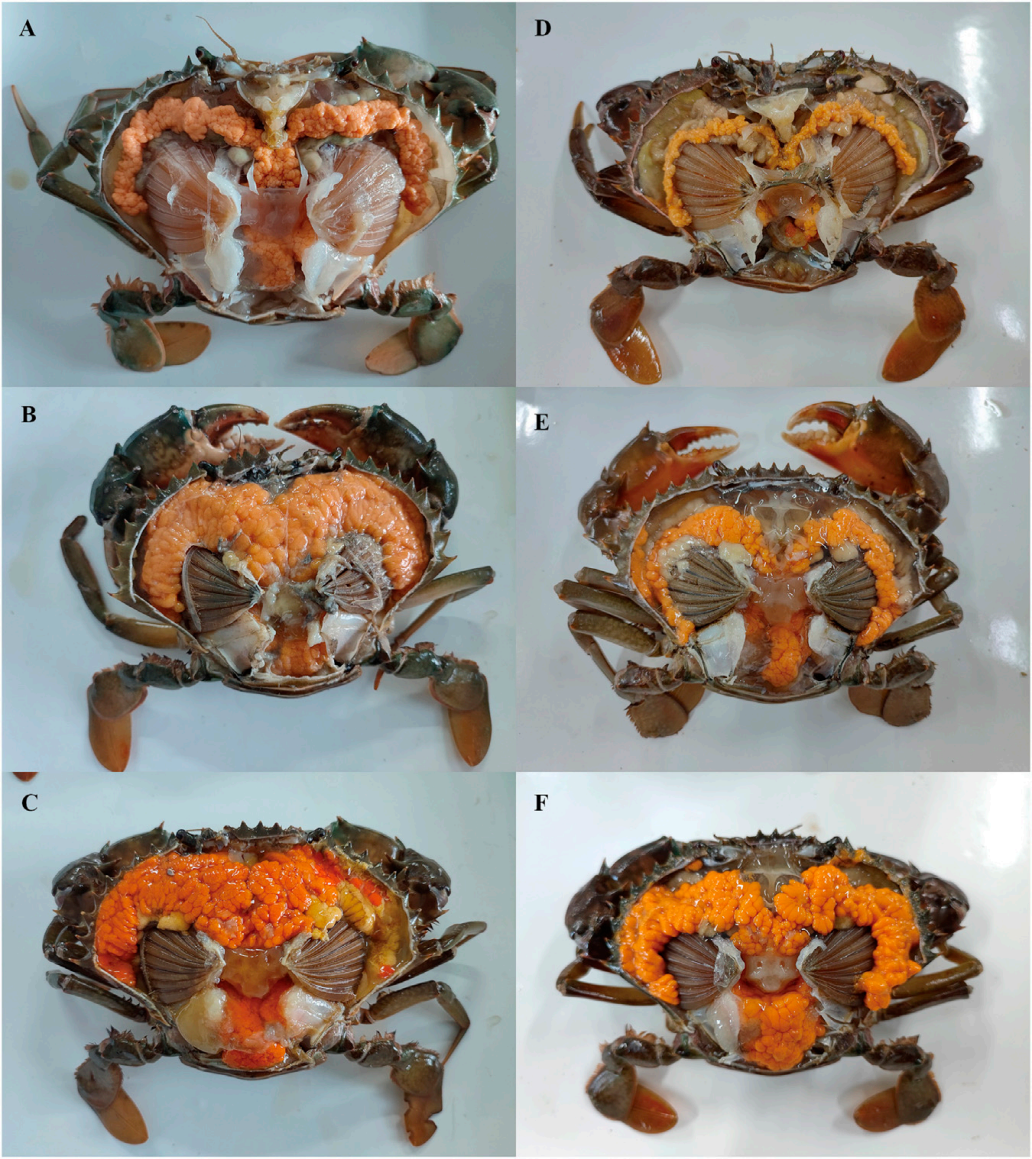
External observation in ovary of *Scylla paramamosain* during the two vitellogenesis periods. **(A)**: ovarian stage Ⅲ of the FVP; **(B)**: ovarian stage Ⅳ of the FVP; **(C)**: ovarian stage Ⅴ of the FVP; **(D)**: ovarian stage Ⅲ of the SVP; **(E)**: ovarian stage Ⅳ of the SVP; **(F)**: ovarian stage Ⅴ of the SVP.

Based on previous studies (Islam et al., 2010; Wu et al., 2020), we determined the ovary of *S. paramamosain* post-spawning to be in ovarian stage Ⅲ. The second ovary maturation period was divided into three stages: vitellogenesis stages (Stage Ⅲ, Ⅳ, and Ⅴ), as shown in Figure 1, Figure 2, and Figure 3, respectively. During the two vitellogenesis periods, many distinctive features, including the ovary appearing light yellow, bright yellow, and bright orange, were identified. Further, the diameters of the oocytes were ∼71.27, ∼127.635, and ∼216 μm in stages Ⅲ, Ⅳ, and Ⅴ, respectively. During the two vitellogenesis periods, the quantity of yolk proteins in oocytes gradually increased.

The diameters of oocytes and hepatic tubules during the two vitellogenesis periods are shown in Table 3. A significant increasing trend was found in oocyte diameter (*p* < 0.05); however, no significant difference was found between the same ovarian stage of the two vitellogenesis periods (*p* > 0.05). In the hepatopancreas, the diameter of the hepatic tubules did not significantly differ during the FVP; however, a significant declining trend was found during the SVP. The diameter of the hepatic tubules during the SVP was also found to be significantly lower than that during the FVP (*p* < 0.05).

**TABLE 3:** The dynamics of oocyte and hepatic tubules diameter during the two vitellogenesis periods in *S. paramamosain*.

Ovarian developmental stages	Oocyte of FVP (μm)	Oocyte of SVP (μm)	Hepatic tubules of FVP (μm)	Hepatic tubules of SVP (μm)
Ⅲ	73.43 ± 2.17^c^	69.11 ± 4.37^c^	297.31 ± 4.04^a^	198.06 ± 8.83^b^
Ⅳ	129.67 ± 0.72^b^	125.60 ± 0.33^b^	289.30 ± 13.39^a^	159.08 ± 29.02^c^
Ⅴ	216.97 ± 5.55^a^	216.50 ± 6.58^a^	270.93 ± 6.85^a^	132.24 ± 7.21^c^

Different superscript letters in the same tissue of the two vitellogenesis periods indicate significant difference (*p* < 0.05).

**FIGURE 4:**
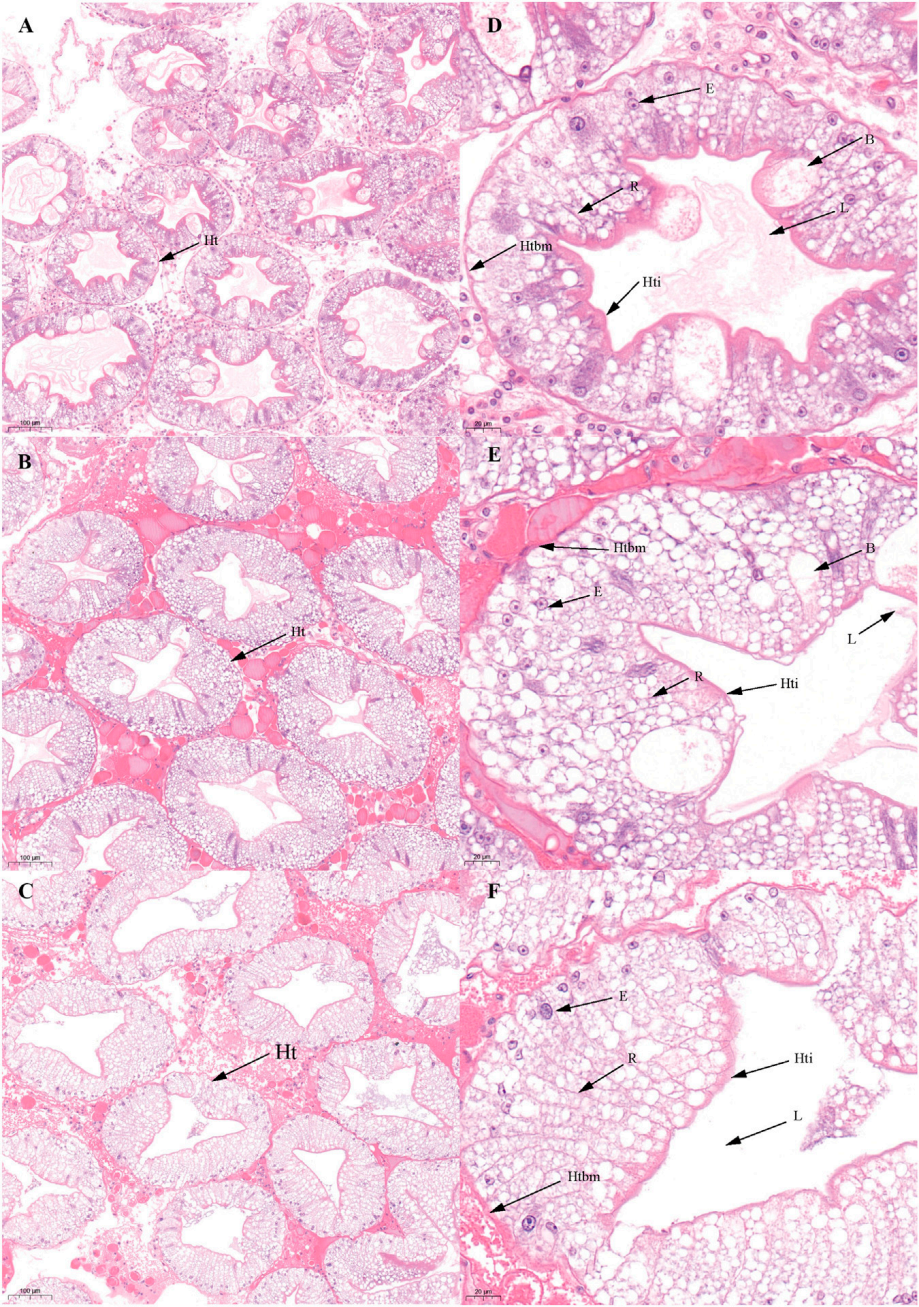
Histological characteristics in the hepatopancreas of *Scylla paramamosain* during the FVP. **(A,D)**: ovarian stage Ⅲ (F-Hep-3); **(B,E)**: ovarian stage Ⅳ (F-Hep-4); **(C,F)**: ovarian stage Ⅴ (F-Hep-5). Ht, Hepatic tubule; B, B-cell; R, R-cell; L, Lumen; Hti, Hepatic tubule intima; Htbm, Hepatic tubule basement membrane.

**FIGURE 5:**
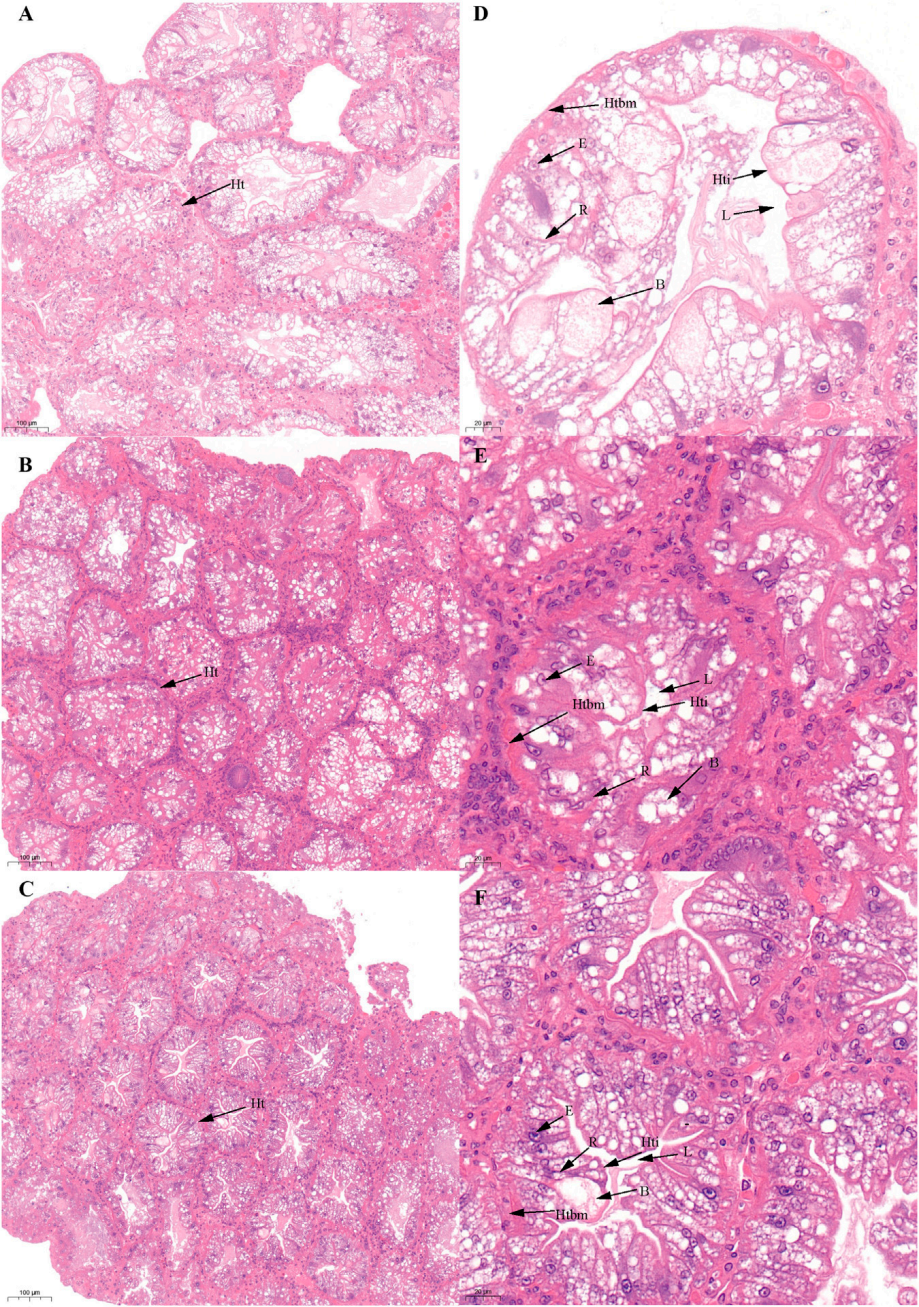
Histological characteristics in the hepatopancreas of *Scylla paramamosain* during the SVP. **(A,D)**: ovarian stage Ⅲ (S-Hep-3); **(B,E)**: ovarian stage Ⅳ (S-Hep-4); **(C,F)**: ovarian stage Ⅴ (S-Hep-5). Ht, Hepatic tubule; B, B-cell; R, R-cell; L, Lumen; Hti, Hepatic tubule intima; Htbm, Hepatic tubule basement membrane.

There was no significant difference in the ovarian histological characteristics between the two vitellogenesis periods (Figure 1 and Figure 2). However, differences were recorded in the external ovarian characteristics, where the ovaries were larger during stage Ⅲ of the FVP than that of the SVP (Figure 3). Notably, during the FVP, many distinctive characteristics, such as a complete structure of the hepatic tubules, a large number of B and R cells, a distinct columnar shape of R cells, many flocculent substances accumulated in the vesicles of B and R cells, and a prominent structure of hepatic tubule lumen were identified in the hepatopancreas (Figure 4). However, during the SVP, the hepatopancreatic tissue displayed many apparent differences, including loose, irregular, and sieve-like shapes of the hepatic tubules, noticeable folds in the intima of the hepatic tubules, cracks or disappearance of the lumen of the hepatic tubules, inconspicuous columnar shape of R cells, and constriction of the connective tissue between adjacent hepatic tubules (Figure 5).

### Dynamic changes in HSI and GSI during the two vitellogenesis periods

**FIGURE 6:**
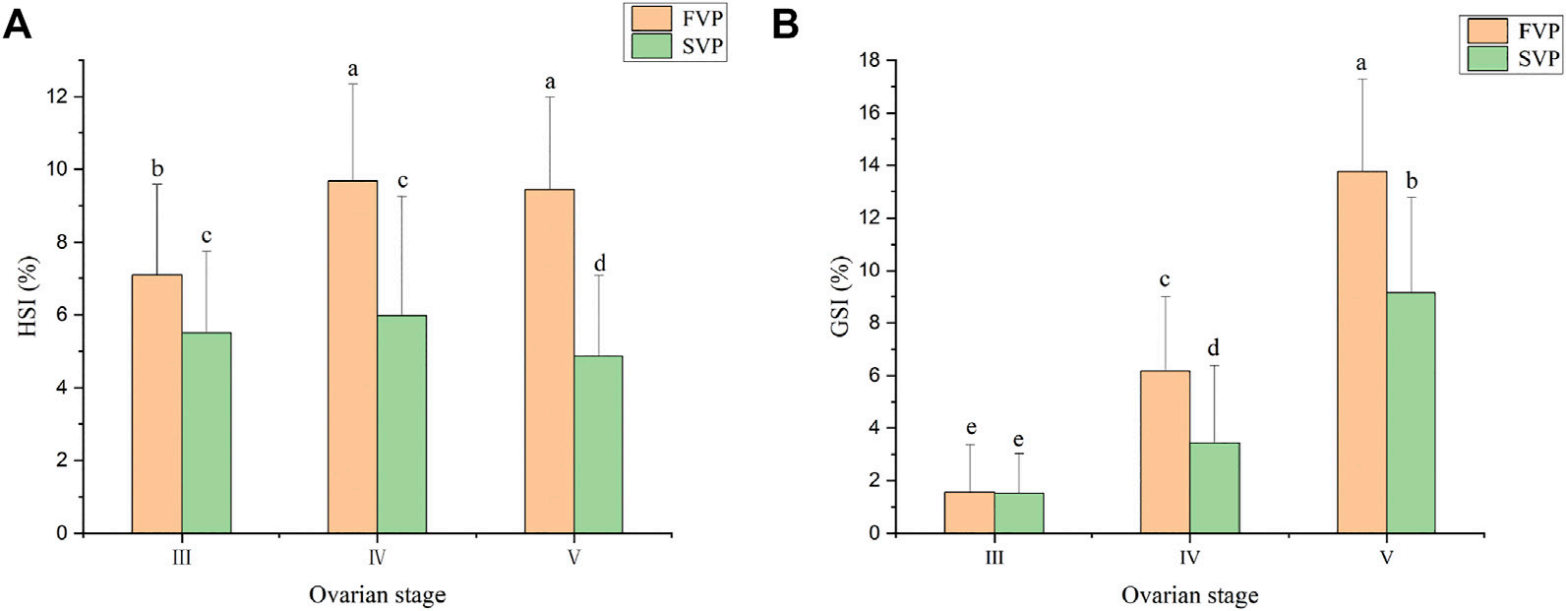
The GSI and HSI of *S. paramamosain* during the two vitellogenesis periods. **(A)** The HSI of *S. paramamosain* during the FVP and SVP. **(B)** The GSI of *S. paramamosain* during the FVP and SVP.

During the two vitellogenesis periods, the GSI displayed a gradually increasing trend (*p* < 0.05) and reached the maximum value (9–14%) in stage Ⅴ. The GSI of the FVP was significantly higher than that of the SVP at the same stage (*p* < 0.05) (Figure 6B).

**FIGURE 7:**
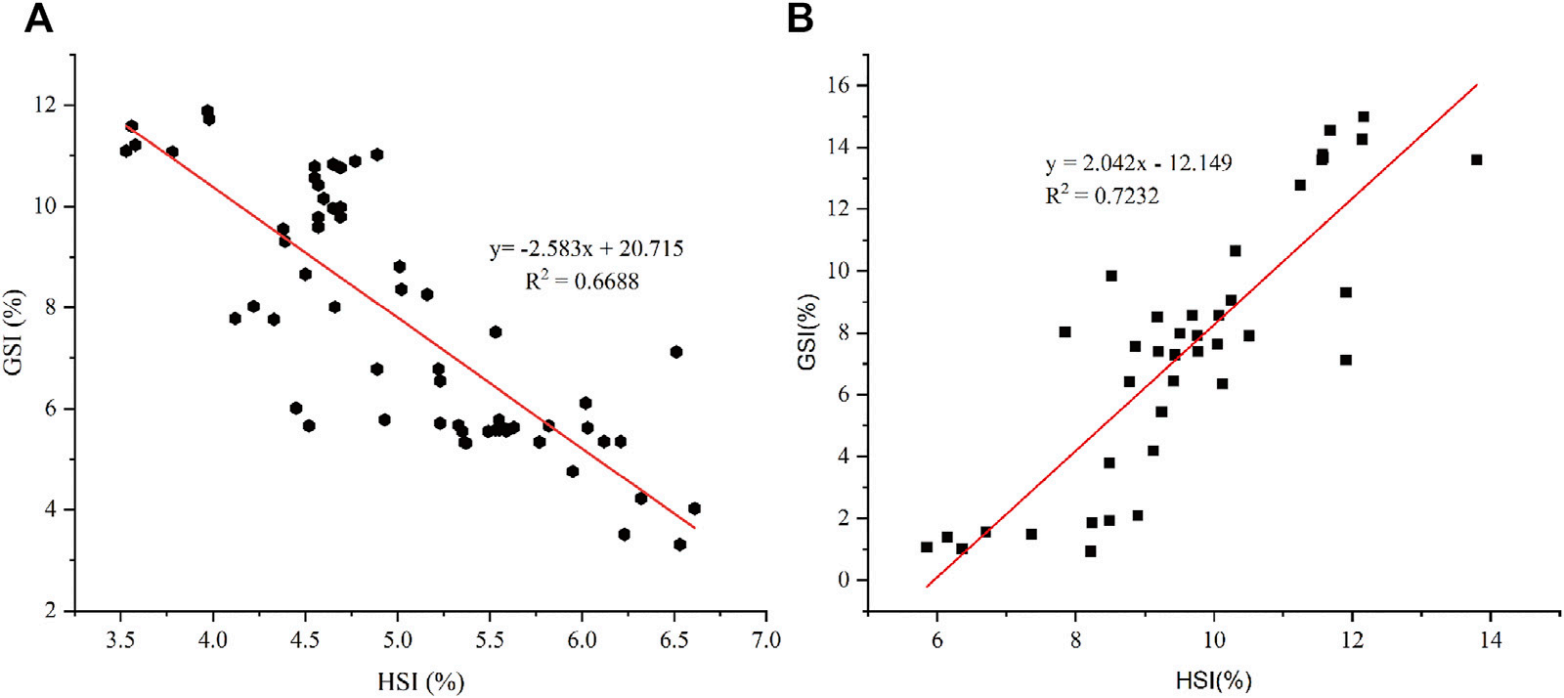
The correlation between HSI and GSI. **(A)** Correlation between HSI and GSI in ovarian stage Ⅳ and Ⅴ during the SVP. **(B)** Correlation between HSI and GSI during the FVP.

The dynamic changes in HSI during the two vitellogenesis periods revealed significant differences, which mainly included two aspects (Figure 6A): 1) the dynamic changes in HSI between the two vitellogenesis periods were approximately opposite; the HSI displayed a significant increasing trend from stage Ⅲ to Ⅳ of the FVP and a significant decreasing trend from stage Ⅳ to Ⅴ of the SVP. 2) Similar to the dynamic GSI changes, the HSI of the FVP at the same stage was higher than that of the SVP. Based on Pearson’s correlation analysis, the GSI from stage Ⅳ to Ⅴ significantly demonstrated a negative correlation with HSI during the SVP (*p* < 0.05) (Figure 7A); however, a significant positive correlation was found between GSI and HSI during the FVP (*p* < 0.05) (Figure 7B).

### Analysis of the *Sp-Vg* expression levels in the hepatopancreas and ovary during the two vitellogenesis periods

**FIGURE 8:**
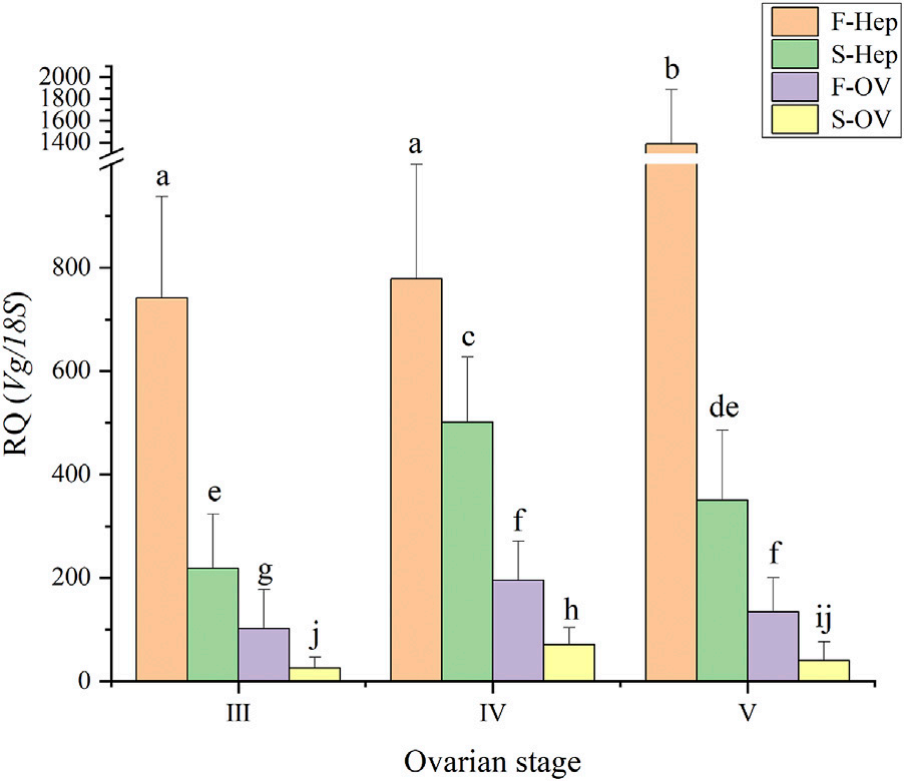
The *Sp-Vg* expression levels in the ovary and hepatopancreas of *Scylla paramamosain* during the two vitellogenesis periods. F-Hep, the hepatopancreas of the FVP; S-Hep, the hepatopancreas of the SVP; F-OV, the ovary of the FVP; S-OV, the ovary of the SVP.

In this study, the expression levels of *Sp-Vg* in the ovary and hepatopancreas during the two vitellogenesis periods were determined (Figure 8). The *Sp-Vg* expression levels in the ovary and hepatopancreas during the FVP were 2–3 fold higher than those during the SVP. In addition, the expression levels of *Sp-Vg* in the hepatopancreas were 2–3 fold higher than those in the ovary during the FVP and SVP.

### Dynamic changes in the proximate composition during the two vitellogenesis periods

The dynamic proximate composition of the hepatopancreas, ovary, and muscle during the two vitellogenesis periods is shown in Table 4. Ovarian protein content displayed an increasing trend between stages Ⅲ and Ⅴ (*p* < 0.05) of the FVP and a significant increasing trend from stage Ⅲ to Ⅴ of the SVP (*p* < 0.05). The ovarian lipid content displayed a slight increase between stages Ⅲ and Ⅳ; however, it significantly increased between stages Ⅲ and Ⅴ of the FVP and significantly declined from stage Ⅳ to Ⅴ during the SVP (*p* < 0.05). In contrast, despite numerous differences between stages Ⅲ and Ⅴ in the two vitellogenesis periods (*p* < 0.05), the ovarian protein content showed an increasing trend. Further, the ovarian protein content significantly increased from stage Ⅳ to Ⅴ and III to V during FVP and SVP (*p* < 0.05), respectively. The lipid content of the hepatopancreas showed a significant increasing trend between stages Ⅲ and Ⅴ of the FVP; however, an initial increasing trend was recorded from stage Ⅲ to Ⅳ, which then significantly decreased from stage Ⅳ to Ⅴ in the SVP (*p* < 0.05). The total protein content of the hepatopancreas remained at the same level during both vitellogenesis periods (*p* < 0.05). The protein content of the muscle during stage Ⅴ of the SVP was significantly higher than that of the FVP; however, the opposite was observed in stage Ⅲ (*p* < 0.05). During the two vitellogenesis periods, the total lipid content of the muscle demonstrated no significant difference from stage Ⅲ to Ⅴ (*p* > 0.05).

**TABLE 4:** The proximate compositions in the ovary, hepatopancreas, and muscle of female *Scylla paramamosain* during the two vitellogenesis periods.

	Moisture (%)	Lipid (%)	Protein (%)	Sugar (%)
F-OV-3	61.77 ± 9.63^b^	9.87 ± 3.79^b^	24.77 ± 5.38^c^	0.90 ± 0.44^b^
F-OV-4	55.93 ± 5.10^b^	12.37 ± 1.75^ab^	27.67 ± 2.12^bc^	1.50 ± 0.60^ab^
F-OV-5	45.07 ± 1.55^c^	15.20 ± 0.76^a^	34.30 ± 0.36^a^	2.10 ± 0.92^a^
S-OV-3	81.7 ± 1.02^a^	2.57 ± 0.15^c^	13.17 ± 0.47^d^	0.60 ± 0.10^b^
S-OV-4	62.27 ± 1.67^b^	8.97 ± 0.41^b^	23.67 ± 1.05^c^	2.17 ± 0.38^a^
S-OV-5	56.3 ± 0.46^b^	10.93 ± 0.65^b^	32.17 ± 1.55^ab^	1.53 ± 0.55^ab^
F-Hep-3	68.60 ± 3.90^b^	18.00 ± 5.00^bc^	10.90 ± 0.72^a^	0.73 ± 0.50^b^
F-Hep-4	60.60 ± 1.78^c^	24.53 ± 6.84^ab^	12.51 ± 4.43^a^	1.67 ± 1.16^ab^
F-Hep-5	52.07 ± 2.745^d^	25.93 ± 3.55^a^	13.40 ± 1.31^a^	2.10 ± 0.46^a^
S-Hep-3	75.5 ± 1.14^a^	8.47 ± 0.51^d^	12.53 ± 0.80^a^	1.53 ± 0.40^ab^
S-Hep-4	69.27 ± 1.19^b^	16.30 ± 1.08^c^	10.70 ± 0.85^a^	0.60 ± 0.10^b^
S-Hep-5	74.77 ± 2.73^a^	9.73 ± 1.00^d^	15.07 ± 1.86^a^	1.50 ± 0.26^ab^
F-Mu-3	74.40 ± 0.98^c^	0.567 ± 0.1528^a^	20.30 ± 0.92^b^	0.1667 ± 0.0577^d^
F-Mu-4	77.17 ± 1.38^bc^	0.553 ± 0.0577^a^	18.83 ± 0.80^bc^	1.400 ± 0.2000^b^
F-Mu-5	77.03 ± 1.30^bc^	0.558 ± 0.1000^a^	19.53 ± 0.764^bc^	0.1667 ± 0.0577^d^
S-Mu-3	84.40 ± 2.26^a^	0.567 ± 0.1528^a^	13.70 ± 0.75^d^	0.800 ± 0.1000^c^
S-Mu-4	78.73 ± 0.97^b^	0.573 ± 0.0577^a^	18.16 ± 1.60^c^	1.900 ± 0.3606^a^
S-Mu-5	76.70 ± 1.21^bc^	0.543 ± 0.1528^a^	22.40 ± 0.98^a^	0.333 ± 0.1155^d^

In the same proximate composition, different superscript letters in the same tissue of the two vitellogenesis period indicate significant difference (*p* < 0.05).

### Dynamic changes in the amino acid composition

During the two vitellogenesis periods, 17 amino acid profiles of the ovary, hepatopancreas, and muscle were identified (Table 5, Table 6, Table 7, respectively). The dominant amino acids in the ovary, hepatopancreas, and muscle are glutamate and aspartate. The total amino acids (TAA) contents in the ovary, hepatopancreas, and muscle displayed an increasing trend between stages Ⅲ and Ⅴ during the two vitellogenesis periods (*p* < 0.05). Further, a rising trend was observed in the ovary and muscle from stage Ⅲ to Ⅴ during the SVP. The TAA contents in the ovary and muscle were significantly higher in stage Ⅲ of the FVP than in that of the SVP (*p* < 0.05).

**TABLE 5:** Profile of amino acids in female *Scylla paramamosain* ovary during the two vitellogenesis periods.

	F-OV-3 (%)	F-OV-4 (%)	F-OV-5 (%)	S-OV-3 (%)	S-OV-4 (%)	S-OV-5 (%)
ASP	2.15 ± 0.61^b^	2.44 ± 0.25^ab^	2.97 ± 0.21^a^	1.24 ± 1.06^c^	2.13 ± 0.11^b^	2.96 ± 0.36^a^
Thr	1.40 ± 0.37^a^	1.55 ± 0.14^a^	1.67 ± 0.28^a^	0.67 ± 0.04^b^	1.45 ± 0.09^a^	1.90 ± 0.37^a^
Ser	1.25 ± 0.49^c^	1.49 ± 0.12^abc^	1.87 ± 0.10^a^	0.56 ± 0.04^d^	0.33 ± 0.09^bc^	1.77 ± 0.31^ab^
Glu	2.92 ± 0.80^a^	3.22 ± 0.34^ab^	3.66 ± 0.49^ab^	1.55 ± 0.08^c^	2.75 ± 0.09^b^	3.78 ± 0.38^a^
Gly	1.11 ± 0.20^a^	1.14 ± 0.13^a^	1.23 ± 0.14^a^	1.32 ± 0.48^a^	1.23 ± 0.13^a^	1.48 ± 0.20^a^
Ala	1.14 ± 0.24^b^	1.17 ± 0.09^b^	1.43 ± 0.07^ab^	0.58 ± 0.03^c^	1.21 ± 0.11^ab^	1.52 ± 0.24^a^
Cys	0.20 ± 0.08^a^	0.16 ± 0.05^a^	0.23 ± 0.03^a^	0.04 ± 0.006^b^	0.21 ± 0.08^a^	0.24 ± 0.08^a^
Val	1.62 ± 0.42^b^	1.74 ± 0.35^ab^	1.92 ± 0.65^ab^	0.65 ± 0.07^c^	1.56 ± 0.11^b^	2.49 ± 0.60^a^
Met	0.97 ± 0.51^bc^	0.75 ± 0.51^cd^	1.63 ± 0.08^a^	0.21 ± 0.04^d^	0.82 ± 0.05^bc^	1.39 ± 0.16^ab^
IIe	1.14 ± 0.34^ab^	1.29 ± 0.30^ab^	1.29 ± 0.51^ab^	0.43 ± 0.12^c^	1.12 ± 0.09^b^	1.72 ± 0.17^a^
Leu	2.08 ± 0.67^b^	2.43 ± 0.32^ab^	3.01 ± 0.35^a^	0.86 ± 0.11^c^	2.11 ± 0.05^b^	2.94 ± 0.49^a^
Tyr	1.11 ± 0.44^b^	1.24 ± 0.09^ab^	1.54 ± 0.09^a^	0.44 ± 0.06^c^	1.19 ± 0.16^ab^	1.32 ± 0.11^ab^
Phe	1.13 ± 0.31^c^	1.28 ± 0.14^bc^	1.63 ± 0.09^a^	0.43 ± 0.05^d^	1.12 ± 0.08^c^	1.43 ± 0.08^ab^
Lys	1.44 ± 0.40^b^	1.78 ± 0.26^ab^	2.07 ± 0.28^a^	0.58 ± 0.04^c^	1.38 ± 0.03^b^	1.96 ± 0.11^a^
His	0.70 ± 0.19^b^	0.78 ± 0.11^ab^	1.07 ± 0.23^a^	0.27 ± 0.07^c^	0.70 ± 0.05^b^	0.89 ± 0.24^ab^
Arg	1.60 ± 0.54^bc^	1.79 ± 0.31^abc^	2.18 ± 0.33^ab^	0.65 ± 0.08^d^	1.54 ± 0.06^c^	2.35 ± 0.23^a^
Pro	1.20 ± 0.27^b^	1.24 ± 0.12^ab^	1.50 ± 0.07^a^	0.64 ± 0.05^c^	1.24 ± 0.10^ab^	1.40 ± 0.10^ab^
EAA	10.48 ± 3.17^bc^	11.60 ± 2.01^abc^	14.28 ± 2.26^ab^	4.10 ± 0.44^d^	10.27 ± 0.31^c^	14.72 ± 2.10^a^
FAA	9.56 ± 2.57^b^	10.48 ± 1.01^ab^	12.45 ± 1.03^a^	5.56 ± 0.51^c^	9.64 ± 0.53^b^	12.50 ± 1.33^a^
TAA	23.17 ± 6.84^b^	25.48 ± 3.40^ab^	30.87 ± 3.69^a^	11.13 ± 0.66^c^	23.10 ± 0.76^b^	31.54 ± 3.98^a^
EAA/TAA	0.452 ± 0.004^a^	0.454 ± 0.019^a^	0.461 ± 0.020^a^	0.368 ± 0.029^b^	0.445 ± 0.005^a^	0.466 ± 0.011^a^
FAA/TAA	0.415 ± 0.011^b^	0.413 ± 0.020^b^	0.405 ± 0.016^b^	0.499 ± 0.034^a^	0.417 ± 0.011^b^	0.397 ± 0.009^b^

In the same amino acid, different superscript letters in the same tissue of the two vitellogenesis period indicate significant difference (*p* < 0.05).

**TABLE 6:** Profile of amino acids in female *Scylla paramamosain* hepatopancreas during the two vitellogenesis periods.

	F-Hep-3 (%)	F-Hep-4 (%)	F-Hep-5 (%)	S-Hep-3 (%)	S-Hep-4 (%)	S-Hep-5 (%)
ASP	1.08 ± 0.09^ab^	1.14 ± 0.25^ab^	1.34 ± 0.25^ab^	1.24 ± 0.25^ab^	0.95 ± 0.09^b^	1.38 ± 0.14^a^
Thr	0.55 ± 0.05^b^	0.57 ± 0.08^b^	0.65 ± 0.09^ab^	0.66 ± 0.12^ab^	0.58 ± 0.09^ab^	0.76 ± 0.10^a^
Ser	0.40 ± 0.04^a^	0.40 ± 0.06^a^	0.42 ± 0.03^a^	0.43 ± 0.08^a^	0.43 ± 0.07^a^	0.45 ± 0.07^a^
Glu	1.17 ± 0.08^a^	1.21 ± 0.28^a^	1.34 ± 0.24^a^	1.30 ± 0.20^a^	1.24 ± 0.13^a^	1.53 ± 0.16^a^
Gly	0.58 ± 0.05^b^	0.58 ± 0.07^b^	0.59 ± 0.07^b^	0.68 ± 0.10^b^	0.65 ± 0.07^b^	0.86 ± 0.11^a^
Ala	0.50 ± 0.04^a^	0.51 ± 0.07^a^	0.52 ± 0.05^a^	0.53 ± 0.05^a^	0.52 ± 0.02^a^	0.60 ± 0.04^a^
Cys	0.05 ± 0.04^a^	0.04 ± 0.02^ab^	0.07 ± 0.14^a^	0.01 ± 0.01^b^	0.43 ± 0.01^ab^	0.06 ± 0.01^a^
Val	0.61 ± 0.04^a^	0.62 ± 0.12^a^	0.49 ± 0.37^a^	0.66 ± 0.11^a^	0.51 ± 0.03^a^	0.75 ± 0.10^a^
Met	0.02 ± 0.005^b^	0.02 ± 0.006^b^	0.06 ± 0.034^a^	0.008 ± 0.007^b^	0.02 ± 0.09^b^	0.28 ± 0.07^ab^
IIe	0.45 ± 0.05^b^	0.48 ± 0.11^ab^	0.55 ± 0.09^ab^	0.48 ± 0.06^ab^	0.46 ± 0.55^ab^	0.64 ± 0.13^a^
Leu	0.82 ± 0.07^a^	0.86 ± 0.14^a^	1.00 ± 0.16^a^	0.83 ± 0.05^a^	0.83 ± 0.08^a^	0.97 ± 0.09^a^
Tyr	0.37 ± 0.14^a^	0.36 ± 0.07^a^	0.49 ± 0.18^a^	0.36 ± 0.07^a^	0.27 ± 0.04^a^	0.45 ± 0.06^a^
Phe	0.50 ± 0.05^ab^	0.50 ± 0.10^ab^	0.60 ± 0.09^ab^	0.56 ± 0.11^ab^	0.43 ± 0.08^b^	0.67 ± 0.10^a^
Lys	0.72 ± 0.07^ab^	0.80 ± 0.14^ab^	0.89 ± 0.09^a^	0.66 ± 0.09^ab^	0.61 ± 0.03^b^	0.77 ± 0.11^ab^
His	0.27 ± 0.03^a^	0.25 ± 0.08^b^	0.41 ± 0.10^ab^	0.25 ± 0.09^b^	0.23 ± 0.08^b^	0.34 ± 0.42^ab^
Arg	0.61 ± 0.10^ab^	0.59 ± 0.11^b^	0.76 ± 0.16^ab^	0.58 ± 0.05^b^	0.61 ± 0.06^ab^	0.80 ± 0.08^a^
Pro	0.49 ± 0.10^ab^	0.48 ± 0.08^b^	0.51 ± 0.07^ab^	0.51 ± 0.06^ab^	0.58 ± 0.04^ab^	0.64 ± 0.06^a^
EAA	3.94 ± 0.33^ab^	4.11 ± 0.74^ab^	4.66 ± 0.71^ab^	4.09 ± 0.43^ab^	3.68 ± 0.22^b^	4.92 ± 0.40^a^
FAA	4.20 ± 0.42^ab^	4.30 ± 0.81^ab^	4.88 ± 0.88^ab^	4.66 ± 0.67^ab^	4.06 ± 0.30^b^	5.48 ± 0.44^a^
TAA	9.19 ± 0.96^ab^	9.41 ± 1.68^ab^	10.69 ± 1.74^ab^	9.71 ± 1.13^ab^	8.98 ± 0.59^b^	11.68 ± 0.85^a^
EAA/TAA	0.429 ± 0.008^ab^	0.426 ± 0.008^a^	0.436 ± 0.015^a^	0.422 ± 0.016^ab^	0.410 ± 0.005^b^	0.421 ± 0.004^ab^
FAA/TAA	0.457 ± 0.002^b^	0.456 ± 0.005^b^	0.456 ± 0.012^b^	0.479 ± 0.016^a^	0.452 ± 0.011^b^	0.469 ± 0.005^ab^

In the same amino acid, different superscript letters in the same tissue of the two vitellogenesis period indicate significant difference (*p* < 0.05).

**TABLE 7:** Profile of amino acids in female *Scylla paramamosain* muscle during the two vitellogenesis periods.

	F-Mu-3 (%)	F-Mu-4 (%)	F-Mu-5 (%)	S-Mu-3 (%)	S-Mu-4 (%)	S-Mu-5 (%)
ASP	2.067 ± 0.0153^abc^	1.943 ± 0.0551^bcd^	2.500 ± 0.5000^a^	1.523 ± 0.1779^d^	1.767 ± 0.1060^cd^	2.300 ± 0.1709^ab^
Thr	0.893 ± 0.0763^bc^	0.910 ± 0.0200^bc^	1.027 ± 0.1650^ab^	0.767 ± 0.0950^c^	0.877 ± 0.1002^bc^	1.223 ± 0.2003^a^
Ser	0.640 ± 0.0265^ab^	0.640 ± 0.0436^ab^	0.727 ± 0.0643^a^	0.530 ± 0.0458^b^	0.660 ± 0.1153^ab^	0.650 ± 0.0889^ab^
Glu	3.057 ± 0.0568^ab^	2.877 ± 0.0850^bc^	2.903 ± 0.3153^bc^	2.327 ± 0.2060^d^	2.537 ± 0.0643^cd^	3.317 ± 0.2203^a^
Gly	1.630 ± 0.0854^b^	1.440 ± 0.0700^b^	2.277 ± 0.2597^a^	1.567 ± 0.1001^b^	1.570 ± 0.0917^b^	1.520 ± 0.1418^b^
Ala	1.243 ± 0.0611^ab^	1.070 ± 0.0625^bc^	1.357 ± 0.1050^a^	0.987 ± 0.1301^c^	1.220 ± 0.1353^ab^	1.383 ± 0.1537^a^
Cys	0.075 ± 0.0053^b^	0.082 ± 0.0036^b^	0.227 ± 0.0681^a^	0.084 ± 0.0095^b^	0.220 ± 0.0900^a^	0.035 ± 0.0074^b^
Val	0.877 ± 0.1026^bc^	0.920 ± 0.7000^ab^	0.770 ± 0.1153^bc^	0.713 ± 0.1002^c^	0.770 ± 0.0985^bc^	1.107 ± 0.1159^a^
Met	0.447 ± 0.0808^b^	0.427 ± 0.0569^b^	0.463 ± 0.1106^b^	0.483 ± 0.1234^a^	0.327 ± 0.0473^b^	0.660 ± 0.1212^a^
IIe	0.883 ± 0.1002^a^	0.863 ± 0.0874^a^	0.620 ± 0.0600^b^	0.577 ± 0.0551^b^	0.610 ± 0.0520^b^	1.047 ± 0.1607^a^
Leu	1.520 ± 0.0755^bc^	1.563 ± 0.0416^bc^	1.683 ± 0.1850^ab^	1.133 ± 0.1193^d^	1.423 ± 0.0850^c^	1.817 ± 0.1484^a^
Tyr	0.620 ± 0.0529^ab^	0.583 ± 0.0306^b^	0.767 ± 0.0404^a^	0.563 ± 0.1106^b^	0.723 ± 0.1050^ab^	0.657 ± 0.1159^ab^
Phe	0.833 ± 0.0643^b^	0.8067 ± 0.0208^b^	0.793 ± 0.0493^bc^	0.623 ± 0.0814^c^	0.767 ± 0.0709^bc^	1.0267 ± 0.1650^a^
Lys	1.647 ± 0.1060^abc^	1.480 ± 0.0854^bc^	1.667 ± 0.1106^ab^	1.207 ± 0.1450^d^	1.450 ± 0.0700^c^	1.837 ± 0.1124^a^
His	0.460 ± 0.0872^ab^	0.377 ± 0.0643^ab^	0.440 ± 0.0916^ab^	0.290 ± 0.0656^b^	0.393 ± 0.1041^ab^	0.517 ± 0.1150^a^
Arg	2.047 ± 0.0702^ab^	1.900 ± 0.0265^ab^	2.067 ± 0.1680^a^	1.307 ± 0.7572^d^	1.693 ± 0.0757^c^	1.867 ± 0.0902^bc^
Pro	1.150 ± 0.0819^bc^	1.453 ± 0.0874^a^	0.823 ± 0.1124^d^	1.000 ± 0.0625^cd^	1.223 ± 0.1150^b^	1.573 ± 0.1007^a^
EAA	7.560 ± 0.2778^b^	7.290 ± 0.1539^b^	7.463 ± 0.7371^b^	5.793 ± 0.7040^c^	6.617 ± 0.3037^bc^	9.233 ± 1.0663^a^
FAA	9.45 ± 0.19^ab^	8.72 ± 0.07^bc^	10.60 ± 1.11^a^	7.59 ± 0.80^d^	8.58 ± 0.31^bc^	10.20 ± 0.82^a^
TAA	20.09 ± 0.37^abc^	19.33 ± 0.16^bc^	21.11 ± 1.91^ab^	15.68 ± 1.54^d^	18.23 ± 0.87^c^	22.54 ± 1.61^a^
EAA/TAA	0.376 ± 0.008^bc^	0.377 ± 0.008^b^	0.353 ± 0.013^c^	0.369 ± 0.010^bc^	0.363 ± 0.004^bc^	0.409 ± 0.020^a^
FAA/TAA	0.470 ± 0.004^b^	0.451 ± 0.002^c^	0.502 ± 0.013^a^	0.484 ± 0.006^b^	0.471 ± 0.007^b^	0.453 ± 0.009^c^

In the same amino acid, different superscript letters in the same tissue of the two vitellogenesis period indicate significant difference (*p* < 0.05).

In the ovary, the contents of flavor amino acids (FAA) and essential amino acids (EAA) were not significantly different from stage Ⅲ to Ⅳ and increased considerably between stages Ⅲ and Ⅴ during the FVP, with a significant increasing trend during the SVP (*p* < 0.05). However, FAA and EAA contents were significantly higher during stage Ⅲ of the FVP than that of the SVP (*p* < 0.05). In the hepatopancreas, the contents of FAA and EAA markedly increased from stage Ⅳ to Ⅴ during the SVP (*p* < 0.05). No significant difference was found between the two vitellogenesis periods in the same stage (*p* > 0.05). In the muscle, the contents of EAA and FAA revealed a significant increasing trend between stages Ⅲ and Ⅴ and were significantly lower in stage Ⅲ of the SVP than that of the SVP. In addition, the FAA content significantly increased from stage Ⅳ to Ⅴ during the FVP (*p* < 0.05).

### Dynamic changes in lipid and fatty acid composition

The fatty acids in the ovary, hepatopancreas, and muscle are shown in Table 8, Table 9, Table 10, respectively. The dominant fatty acids in the ovary and hepatopancreas were C16:0 and C18:1n9c, respectively. No significant difference was observed in the content of HUFA and DHA in the ovary from stage Ⅲ to Ⅴ during the two vitellogenesis periods; however, the content of HUFA was higher in stage Ⅴ of the FVP than that of the SVP. The SFA content displayed a significant increasing trend between stages Ⅲ and Ⅴ during the two vitellogenesis periods. Further, a significant increasing trend was found in the MUFA content between stages Ⅲ and Ⅴ of the FVP and Ⅲ to Ⅴ of the SVP.

**TABLE 8:** Profile of fatty acids in female *Scylla paramamosain* ovary during the two vitellogenesis periods.

	F-OV-3 (%)	F-OV-4 (%)	F-OV-5 (%)	S-OV-3 (%)	S-OV-4 (%)	S-OV-5 (%)
C12:0	0.017 ± 0.0019^b^	0.016 ± 0.0019^b^	0.264 ± 0.0078^a^	0^c^	0.024 ± 0.0059^a^	0.026 ± 0.0098^a^
C13:0	0.007 ± 0.0004^b^	0.007 ± 0.013^b^	0.013 ± 0.0028^a^	0^c^	0.008 ± 0.0004^b^	0.007 ± 0.0042^b^
C14:0	0.132 ± 0.0446^a^	0.148 ± 0.021^a^	0.186 ± 0.0677^a^	0.161 ± 0.2068^a^	0.161 ± 0.0942^a^	0.165 ± 0.1332^a^
C15:0	0.046 ± 0.0062^bc^	0.756 ± 0.050^ab^	0.104 ± 0.050^a^	0.017 ± 0.0027^c^	0.074 ± 0.0111^ab^	0.063 ± 0.0052^ab^
C16:0	1.449 ± 0.7905^bc^	1.819 ± 0.4852^ab^	2.354 ± 0.5237^a^	0.6102 ± 0.0279^c^	1.631 ± 0.0742^ab^	1.859 ± 0.0693^ab^
C16:1	0.433 ± 0.2015^bc^	0.522 ± 0.1213^bc^	1.148 ± 0.0826^a^	0.112 ± 0.0817^d^	0.385 ± 0.0429^c^	0.648 ± 0.1316^b^
C17:0	0.064 ± 0.0245^b^	0.081 ± 0.0176^b^	0.125 ± 0.0123^a^	0.041 ± 0.0388^b^	0.082 ± 0.0095^b^	0.083 ± 0.0085^ab^
C18:0	0.553 ± 0.3198^bc^	0.786 ± 0.2498^ab^	1.023 ± 0.2605^a^	0.188 ± 0.0101^c^	0.637 ± 0.0438^ab^	0.798 ± 0.0543^ab^
C18:1n9c	1.188 ± 0.6194^b^	1.376 ± 0.4029^ab^	1.935 ± 0.1897^a^	0.325 ± 0.0332^c^	0.857 ± 0.0531^bc^	1.071 ± 0.1325^b^
C18:2n6c	0.170 ± 0.0802^b^	0.354 ± 0.1791^b^	0.959 ± 0.0801^a^	0.039 ± 0.0041^b^	0.177 ± 0.0098^b^	0.313 ± 0.4215^b^
C20:0	0.043 ± 0.0142^c^	0.049 ± 0.0131^bc^	0.076 ± 0.0112^a^	0.020 ± 0.0015^d^	0.064 ± 0.0080^ab^	0.071 ± 0.0058^a^
C18:3n6	0.006 ± 0.0052^c^	0.009 ± 0.0020^bc^	0.0142 ± 0.0025^a^	0^d^	0.013 ± 0.0003^ab^	0.008 ± 0.0010^c^
C18:3n3	0.039 ± 0.0179^b^	0.061 ± 0.0399^b^	0.232 ± 0.0698^a^	0.021 ± 0.0018^b^	0.062 ± 0.0036^b^	0.029 ± 0.0083^b^
C20:1	0.058 ± 0.0240^b^	0.073 ± 0.0188^b^	0.100 ± 0.0442^ab^	0.037 ± 0.0068^b^	0.076 ± 0.0086^b^	0.152 ± 0.0706^a^
C21:0	0.011 ± 0.0030^a^	0.013 ± 0.0024^a^	0.030 ± 0.0029^a^	0.007 ± 0.0008^a^	0.027 ± 0.0013^a^	0.181 ± 0.2473^a^
C20:2	0.054 ± 0.0126^b^	0.065 ± 0.0193^b^	0.182 ± 0.0729^a^	0.037 ± 0.0053^b^	0.082 ± 0.0033^b^	0.068 ± 0.0095^b^
C22:0	0.026 ± 0.0074^b^	0.268 ± 0.0075^b^	0.0478 ± 0.0037^a^	0.009 ± 0.0008^d^	0.0303 ± 0.0046^b^	0.365 ± 0.0045^b^
C20:3n6	0.144 ± 0.0027^cd^	0.164 ± 0.0028^cd^	0.356 ± 0.0063^a^	0.007 ± 0.0010^d^	0.028 ± 0.0045^ab^	0.022 ± 0.0110^bc^
C20:3n3	0.024 ± 0.0047^b^	0.025 ± 0.0121^ab^	0.065 ± 0.0122^a^	0.020 ± 0.0034^b^	0.041 ± 0.0029^a^	0.029 ± 0.0078^ab^
C20:4n6	0.279 ± 0.1038^a^	0.199 ± 0.1305^a^	0.316 ± 0.2295^a^	0.153 ± 0.0408^a^	0.308 ± 0.0167^a^	0.100 ± 0.0126^a^
C22:1n9	0.031 ± 0.0053^ab^	0.034 ± 0.0079^ab^	0.042 ± 0.0124^ab^	0.026 ± 0.0027^b^	0.047 ± 0.0117^a^	0.036 ± 0.0084^ab^
C23:0	0.008 ± 0.0025^cd^	0.0086 ± 0.0021^cd^	0.019 ± 0.0034^ab^	0^d^	0.012 ± 0.0018^bc^	0.021 ± 0.0096^a^
C20:5n3 (EPA)	0.569 ± 0.2558^ab^	0.379 ± 0.3608^bc^	0.848 ± 0.1274^a^	0.189 ± 0.0102^c^	0.4701 ± 0.04020^abc^	0.177 ± 0.0129^c^
C24:0	0.024 ± 0.0125^b^	0.027 ± 0.0055^b^	0.0395 ± 0.0137^ab^	0^c^	0.028 ± 0.0054^ab^	0.045 ± 0.1002^a^
C24:1	0.026 ± 0.0127^a^	0.028 ± 0.0109^a^	0.023 ± 0.0071^a^	0.005 ± 0.0002^b^	0.019 ± 0.0031^ab^	0.019 ± 0.0009^ab^
C22:6n3 (DHA)	1.153 ± 0.8521^a^	0.659 ± 0.7915^a^	0.626 ± 0.6309^a^	0.146 ± 0.0076^a^	0.693 ± 0.0719^a^	0.171 ± 0.0143^a^
HUFA	2.340 ± 1.1132^ab^	1.802 ± 1.4649^ab^	3.320 ± 0.9258^a^	0.637 ± 0.0228^b^	0.821 ± 0.1026^b^	0.953 ± 0.4888^b^
SFA	2.38 ± 1.22^bc^	3.06 ± 0.72^ab^	4.04 ± 0.89^a^	1.05 ± 0.24^c^	2.74 ± 0.04^ab^	3.36 ± 0.35^ab^
MUFA	0.52 ± 0.23^c^	0.62 ± 0.10^bc^	1.27 ± 0.12^a^	0.15 ± 0.08^d^	0.48 ± 0.04^c^	0.82 ± 0.20^b^

In the same fatty acid, different superscript letters in the same tissue of the two vitellogenesis period indicate significant difference (*p* < 0.05).

**TABLE 9:** Profile of fatty acids in female *Scylla paramamosain* hepatopancreas during the two vitellogenesis periods.

	F-Hep-3 (%)	F-Hep-4 (%)	F-Hep-5 (%)	S-Hep-3 (%)	S-Hep-4 (%)	S-Hep-5 (%)
C12:0	0.02 ± 0.004^b^	0.03 ± 0.007^ab^	0.04 ± 0.009^a^	0.01 ± 0.002^c^	0.02 ± 0.001^bc^	0.02 ± 0.012^b^
C13:0	0.01 ± 0.004^abc^	0.02 ± 0.012^ab^	0.03 ± 0.013^a^	0^c^	0.01 ± 0.001^abc^	0.007 ± 0.001^bc^
C14:0	0.44 ± 0.12^a^	0.59 ± 0.33^a^	0.51 ± 0.15^a^	0.08 ± 0.009^b^	0.37 ± 0.05^ab^	0.29 ± 0.04^ab^
C15:0	0.15 ± 0.09^a^	0.30 ± 0.36^a^	0.27 ± 0.06^a^	0.04 ± 0.01^a^	0.31 ± 0.02^a^	0.09 ± 0.01^a^
C16:0	3.21 ± 1.03^bc^	4.37 ± 1.08^ab^	4.92 ± 0.51^a^	1.24 ± 0.12^d^	3.56 ± 0.36^bc^	2.41 ± 0.14^cd^
C16:1	1.07 ± 0.39^b^	1.31 ± 0.76^b^	2.19 ± 0.52^a^	0.19 ± 0.02^c^	0.86 ± 0.06^bc^	0.76 ± 0.12^bc^
C17:0	0.13 ± 0.04^bc^	0.17 ± 0.06^bc^	0.18 ± 0.02^b^	0.15 ± 0.04^bc^	0.30 ± 0.06^a^	0.09 ± 0.01^c^
C18:0	0.95 ± 0.39^abc^	1.19 ± 0.43^ab^	1.18 ± 0.06^ab^	0.64 ± 0.12^bc^	1.31 ± 0.17^a^	0.52 ± 0.38^c^
C18:1n9c	2.07 ± 0.39^ab^	3.1 ± 0.86^a^	3.03 ± 1.45^a^	0.68 ± 0.09^b^	1.46 ± 0.12^b^	1.34 ± 0.20^b^
C18:2n6c	0.39 ± 0.31^b^	1.07 ± 0.31^a^	1.16 ± 0.64^a^	0.26 ± 0.06^b^	0.40 ± 0.02^b^	0.34 ± 0.12^b^
C20:0	0.09 ± 0.03^a^	0.11 ± 0.04^a^	0.13 ± 0.001^a^	0.08 ± 0.007^a^	0.13 ± 0.011^a^	0.11 ± 0.04^a^
C18:3n6	0.015 ± 0.008^bc^	0.03 ± 0.02^ab^	0.02 ± 0.006^ab^c	0.008 ± 0.0004^c^	0.035 ± 0.01^a^	0.008 ± 0.0009^c^
C18:3n3	0.09 ± 0.07^b^	0.20 ± 0.18^ab^	0.33 ± 0.18^a^	0.056 ± 0.01^b^	0.22 ± 0.09^ab^	0.04 ± 0.01^b^
C20:1	0.11 ± 0.05^a^	0.18 ± 0.06^a^	0.24 ± 0.10^a^	0.21 ± 0.09^a^	0.18 ± 0.01^a^	0.16 ± 0.03^a^
C21:0	0.03 ± 0.008^d^	0.05 ± 0.01^cd^	0.06 ± 0.008^abc^	0.07 ± 0.01^ab^	0.08 ± 0.008^a^	0.05 ± 0.009^bcd^
C20:2	0.08 ± 0.03^b^	0.15 ± 0.07^ab^	0.23 ± 0.05^a^	0.19 ± 0.01^a^	0.18 ± 0.03^a^	0.08 ± 0.008^b^
C22:0	0.08 ± 0.03^b^	0.10 ± 0.02^b^	0.11 ± 0.02^ab^	0.07 ± 0.01^b^	0.21 ± 0.10^a^	0.08 ± 0.009^b^
C20:3n6	0.02 ± 0.007^c^	0.05 ± 0.03^ab^	0.03 ± 0.003^bc^	0.02 ± 0.005^bc^	0.07 ± 0.01^a^	0.02 ± 0.002^c^
C20:3n3	0.04 ± 0.01^b^	0.05 ± 0.03^b^	0.10 ± 0.009^a^	0.06 ± 0.008^b^	0.09 ± 0.009^a^	0.03 ± 0.007^b^
C20:4n6	0.25 ± 0.05^ab^	0.30 ± 0.16^ab^	0.31 ± 0.07^a^	0.25 ± 0.02^ab^	0.40 ± 0.08^a^	0.14 ± 0.02^b^
C22:1n9	0.03 ± 0.01^a^	0.04 ± 0.02^a^	0.03 ± 0.004^a^	0.05 ± 0.01^a^	0.05 ± 0.008^a^	0.05 ± 0.02^a^
C23:0	0.03 ± 0.008^b^	0.03 ± 0.005^ab^	0.06 ± 0.02^a^	0.03 ± 0.009^ab^	0.04 ± 0.006^ab^	0.03 ± 0.009^ab^
C20:5n3 (EPA)	0.44 ± 0.11^a^	0.43 ± 0.12^a^	0.34 ± 0.25^a^	0.29 ± 0.05^a^	0.30 ± 0.04^a^	0.19 ± 0.02^a^
C24:0	0.09 ± 0.05^a^	0.10 ± 0.04^a^	0.10 ± 0.02^a^	0.07 ± 0.02^a^	0.14 ± 0.06^a^	0.08 ± 0.009^a^
C24:1	0.09 ± 0.07^a^	0.12 ± 0.08^a^	0.04 ± 0.01^a^	0.02 ± 0.009^a^	0.08 ± 0.01^a^	0.08 ± 0.01^a^
C22:6n3 (DHA)	0.90 ± 0.61^a^	0.71 ± 0.29^a^	0.53 ± 0.27^a^	0.45 ± 0.10^a^	0.59 ± 0.09^a^	0.36 ± 0.07^a^
HUFA	2.25 ± 0.37^ab^	3.04 ± 0.83^a^	3.06 ± 0.59^a^	1.64 ± 0.25^bc^	2.32 ± 0.22^ab^	1.25 ± 0.27^c^
SFA	5.24 ± 1.70^c^	7.05 ± 1.92^ab^	7.60 ± 0.43^a^	2.48 ± 0.33^d^	6.49 ± 0.11^ab^	3.78 ± 0.19^cd^
MUFA	1.27 ± 0.41^b^	1.60 ± 0.72^b^	2.46 ± 0.41^a^	0.42 ± 0.12^c^	1.11 ± 0.08^bc^	0.99 ± 0.16^bc^

In the same fatty acid, different superscript letters in the same tissue of the two vitellogenesis period indicate significant difference (*p* < 0.05).

**TABLE 10:** Profile of fatty acids in female *Scylla paramamosain* muscle during the two vitellogenesis periods.

	F-Mu-3 (%)	F-Mu-4 (%)	F-Mu-5 (%)	S-Mu-3 (%)	S-Mu-4 (%)	S-Mu-5 (%)
C16:0	0.078 ± 0.0100^a^	0.009 ± 0.0006^a^	0.036 ± 0.0066^a^	0.027 ± 0.0098^a^	0.179 ± 0.2479^a^	0.036 ± 0.0061^a^
C16:1	0.015 ± 0.0050^a^	0^b^	0.006 ± 0.0014^b^	0.006 ± 0.0012^b^	0.017 ± 0.0054^a^	0.019 ± 0.0071^a^
C17:0	0.024 ± 0.0302^a^	0^a^	0^a^	0^a^	0^a^	0^a^
C18:0	0.071 ± 0.0097^a^	0.021 ± 0.0007^d^	0.047 ± 0.0105^b^c	0.033 ± 0.0037^cd^	0.053 ± 0.0113^b^	0.047 ± 0.0035^bc^
C18:1n9c	0.085 ± 0.0045^a^	0.016 ± 0.0010^c^	0.047 ± 0.0107^b^	0.035 ± 0.0115^b^	0.043 ± 0.0070^b^	0.047 ± 0.0084^b^
C18:2n6c	0.012 ± 0.0008^ab^	0.004 ± 0.0002^c^	0.008 ± 0.0006^bc^	0.006 ± 0.0012^c^	0.016 ± 0.0057^a^	0.007 ± 0.0014^bc^
C18:3n3	0^c^	0^c^	0^c^	0.005 ± 0.0014^b^	0.007 ± 0.0010^a^	0^c^
C20:1	0.005 ± 0.0005^a^	0^b^	0^b^	0^b^	0^b^	0^b^
C20:4n6	0.015 ± 0.0012^c^	0^d^	0.013 ± 0.0043^cd^	0.027 ± 0.0076^bc^	0.056 ± 0.115^a^	0.035 ± 0.0107^b^
C22:1n9	0.006 ± 0.0003^c^	0.012 ± 0.0028^abc^	0.009 ± 0.0032^bc^	0.024 ± 0.0092^a^	0.021 ± 0.0119^ab^	0.005 ± 0.0009^c^
C20:5n3 (EPA)	0.041 ± 0.0021^bc^	0.005 ± 0.0006^d^	0.035 ± 0.0113^c^	0.033 ± 0.0050^c^	0.067 ± 0.0117^a^	0.060 ± 0.0164^ab^
C22:6n3 (DHA)	0.043 ± 0.0030^a^	0.007 ± 0.0005^b^	0.028 ± 0.0070^a^	0.036 ± 0.0157^a^	0.040 ± 0.0051^a^	0.031 ± 0.0049^a^
HUFA	0.117 ± 0.0053^b^	0.028 ± 0.0023^c^	0.094 ± 0.0243^b^	0.131 ± 0.0370^b^	0.213 ± 0.0426^a^	0.143 ± 0.0339^b^
SFA	0.173 ± 0.023^a^	0.029 ± 0.01^a^	0.084 ± 0.017^a^	0.060 ± 0.011^a^	0.232 ± 0.259^a^	0.083 ± 0.009^a^
MUFA	0.020 ± 0.005^a^	0^b^	0.006 ± 0.001^b^	0.006 ± 0.001^b^	0.017 ± 0.005^a^	0.019 ± 0.007^a^

In the same fatty acid, different superscript letters in the same tissue of the two vitellogenesis period indicate significant difference (*p* < 0.05).

A significant increasing trend in the SFA content of the hepatopancreas was found from stage Ⅲ to Ⅴ of the FVP and Ⅳ to Ⅴ of the SVP. The MUFA content significantly increased between stages Ⅳ and Ⅴ during the FVP; however, no significant difference was observed during the SVP. In addition, the HUFA, SFA, and MUFA contents were not significantly different in stage Ⅳ between the two vitellogenesis periods; however, the SFA and MUFA contents were significantly higher in stage Ⅲ of the FVP than that of the SVP (*p* < 0.05).

In the muscle, the DHA content displayed a parabolic shape from stage Ⅲ to Ⅴ of the FVP, while the HUFA content displayed a U shape during the FVP and vice versa during the SVP. The DHA and HUFA contents in stage Ⅳ of the FVP were significantly higher than those of the SVP; however, the MUFA content in stage Ⅴ of the SVP was significantly higher than that of the FVP.

## Discussion

### Morphology, HSI, and GSI analysis

Based on the findings of the present study, external and histological changes were initially demonstrated to occur during the second ovarian development period, including ovarian stages Ⅲ, Ⅳ, and Ⅴ (vitellogenesis period), after spawning. Further, the ovary during the two vitellogenesis periods of *S. paramamosain* appeared light yellow in ovarian stage Ⅲ, bright yellow in ovarian stage Ⅳ, and bright orange in ovarian stage Ⅴ. The oocyte diameter displayed a significant increasing trend in the vitellogenesis period, and the yolk granules in the cytoplasm of oocytes gradually increased, similar to the results of a previous study (Wu et al., 2020). Notably, in the present study, no significant differences were initially found in the external and histological characteristics of the ovary between the two vitellogenesis periods, including changes in the coloration of the ovary and changes in oocyte size during the same ovarian stage of the two vitellogenesis periods. Such a finding indicates that the ovaries of *S. paramamosain* during the SVP can re-develop and achieve the state of first ovarian maturation, similar to the findings of a previous study (Yu et al., 2021). However, the size of the ovary in stage Ⅲ of the FVP was larger than that of the SVP, which might be due to the decrease in the number of oocytes after spawning. In addition, there were many noticeable differences in the hepatopancreas between the two vitellogenesis periods. To the best of our knowledge, this is the first study to reveal the distinct characteristics of the hepatopancreas during the two vitellogenesis periods. Similar to a previous study (Cheng et al., 2000), many obvious features of the hepatic tubules such as a loose and sieve-like appearance, a gradually decreasing trend in the diameter of the hepatic tubules, shrinkage of the lumen, and gradual adherence of the intima were recognized during the SVP. This observation indicates that the hepatopancreas degenerated during the SVP, which was significantly different from that observed in the hepatopancreas during the FVP, ultimately implying that significant differences in the provision of nutrients by the hepatopancreas may exist during the two ovarian maturation periods.

The HSI provides an indication of nutrient storage in the hepatopancreas (Chellappa et al., 1995), and the GSI is an indicator of the development and maturation status of the gonadal tissue (Flores et al., 2015). The nutrients required for ovarian development are synthesized and transported by the hepatopancreas, and the HSI gradually decreases as the GSI increases (Chu, 1999; Yu et al., 2007). However, based on previous research (Wu et al., 2020) using *S. paramamosain*, some distinct features were identified as the nutrients were accumulated and transported from the hepatopancreas to the ovary. The HSI was not negatively correlated with GSI during the period of ovary development. The results obtained in the present study during the FVP were similar to those obtained in a previous study (Wu et al., 2020); however, the characteristic changes in the hepatopancreas during the FVP did not apply to those during the SVP. Accordingly, we speculate that the method of nutritional accumulation and transportation in the hepatopancreas during SVP differs from that during SVP in *S. paramamosain*, especially in terms of nutrient transport from the hepatopancreas to the ovary.

### Analysis of the *Sp-Vg* expression levels during the two vitellogenesis periods

In crustaceans, vitellogenesis, also known as the accumulation of Vg or vitellin (Vn) in oocytes, is a key event in ovarian maturation. Vitellin, the major yolk protein produced by a precursor protein called Vg, provides proteins, carbohydrates, lipids, and other nutrients to maturing oocytes as resources for embryonic development (Valle, 1993; Chen et al., 1997) and combines with metallic ions such as Zn^2+^, Fe^2+^, Cu^2+^, Mg^2+^, and Ca^2+^ and transports them to oocytes (Taborsky, 1980; Ghosh & Thomas, 1995; Montorzi et al., 1995). In addition, carotenoids, thyroxine, retinol, and riboflavin can be transported to oocytes during vitellogenesis by a Vg carrier (Ando & Hatano, 1991; Babin, 1992; Azuma et al., 1993; Mac Lachlan et al., 1994). The nutrients carried by Vg are crucial for the growth of larvae before self-feeding (Subramoniam, 2011; Jia et al., 2013). The hepatopancreas and ovary are the primary tissues where *Sp-Vg* is predominantly expressed in *S. paramamosain* (Li et al., 2006; Jia et al., 2013). To our knowledge, this is the first study to characterize *Sp-Vg* expression levels in the hepatopancreas and ovary of *S. paramamosain* during the two vitellogenesis periods. One of the most striking features was the hepatopancreas serving as the dominant tissue for *Sp-Vg* expression, similar to previous findings for the first ovarian maturation (Yang et al., 1996; Yang et al., 2005). Notably, significantly different from previous studies during FVP (Jia et al., 2013), the expression levels of *Sp-Vg* in the hepatopancreas and ovary displayed a significant decreasing trend from ovarian stage Ⅳ to Ⅴ of the SVP in the present study, indicating that the capacity for Vg synthesis gradually decreased during the SVP. Such a finding suggests that the two vitellogenesis periods were mainly dependent on the hepatopancreas for the provision of nutrients, and the expression levels of *Sp-Vg* in the hepatopancreas and ovary during SVP were significantly lower than those during FVP, which implied that the capacity of Vg synthesis during SVP was distinctly lower than that during FVP.

### Analysis of the total protein and amino acid composition of various tissues

In this study, the total protein and amino acids were profiled, representing two indicators of the nutritional value of the edible parts of *S. paramamosain.* The predominant components in the ovary and muscle during the two vitellogenesis periods were mainly proteins while those in the hepatopancreas were mainly lipids, aligning with the findings of previous studies (Jiang et al., 2014; Wu et al., 2020). In the ovary and hepatopancreas, the total protein, EAA/TAA, and FAA/TAA, were not significantly different between the two vitellogenesis periods. Based on the ideal value of EAA/TAA (0.4) in foods recommended by the FAO/WHO/UNU (1985), the value of EAA/TAA in the hepatopancreas and ovary during the two vitellogenesis periods, except for ovary in stage Ⅲ during the SVP, exceeded 0.4. From the perspective of protein nutritional value, this finding indicated that the hepatopancreas and ovary can not only meet the demands of food nutritional value during the two vitellogenesis periods (except the ovary of stage Ⅲ during the SVP) but can also achieve the nutritional status of the first ovarian maturation, similar to the finding of a previous study (Yu et al., 2021). According to our research, the total protein and the value of EAA/TAA in the ovary were the lowest in stage Ⅲ of the SVP, while the value of FAA/TAA was the highest, indicating that the nutrients in the ovarian tissue may be mainly used for embryonic development and self-nutrient supply, resulting in a significant decreasing trend for the EAA/TAA value of ovaries after spawning and a relative increase in the corresponding FAA/TAA. Notably, the total protein and the value of EAA/TAA in the muscle were significantly higher in SVP than in FVP; however, the value of EAA/TAA in stage Ⅴ of the SVP was substantially lower than that of the FVP, suggesting that the nutritional value of the muscle during the second ovarian maturation might be higher than that of the first ovarian maturation. We speculated that numerous nutrients must be rapidly synthesized and transported to the ovary to meet the nutrient requirement owing to a shorter second ovarian maturation period. Notably, a large quantity of nutrients was consumed during the first brooding period.

### Analysis of the total lipid and fatty acid composition in various tissues

Lipids are not only important nutrients in the hepatopancreas and ovary but also an essential nutritional basis for ovarian maturation (Harrison, 1990; Iqbal et al., 2006; Alava et al., 2007). Based on our study, the dominant fatty acids in the hepatopancreas and ovary were C16:0 and C18:1n9c, respectively. The lipid content in the ovary and hepatopancreas displayed an overall increasing trend during the FVP, and the content of fatty acids in the muscle did not significantly differ between the two vitellogenesis periods, thereby aligning with the findings of previous studies (Jiang et al., 2014; Wu et al., 2020). During the SVP, the lipid content in the ovary mainly increased; however, in the hepatopancreas, the lipid content initially increased and then decreased, which differed from the results obtained in the FVP in a previous study (Wu et al., 2020). Such a finding implies that some differences may exist in the transport patterns of nutrients between the two vitellogenesis periods.

During the two vitellogenesis periods, HUFA, SFA, and MUFA in the ovary displayed an increasing trend, which implied that these fatty acids were not only important energy substances but also necessary nutrients for ovarian maturation and embryonic development (Ying et al., 2006). Based on previous studies (Yang et al., 1996; Reppond et al., 2009), EPA and DHA are vital nutrients for the development of reproductive function in crustaceans, especially the formation of egg stalks and ovulation after the maturation of the ovary. In this study, there was no significant difference in the content of ovarian DHA during the two vitellogenesis periods; however, the contents of ovarian EPA, MUFA, and HUFA in stage Ⅴ of the SVP were significantly lower than those of the FVP, which indicates that the abortion rate of *S. paramamosain* during the SVP may be higher than that during the FVP. According to a previous study (Yu et al., 2021), the egg-holding rate of the second ovarian maturation of *S. paramamosain* was significantly lower than that of the first ovarian maturation. Therefore, we speculated that the decrease in EPA, MUFA, and HUFA content might be a key reason for the decline in the egg-holding rate of *S. paramamosain* during the SVP.

Previous studies have shown that the synthetic capacity of lipids in the hepatopancreas during the period of ovarian development was considerably stronger than that in the ovary owing to the reason that a considerable part of the lipids must be transferred from the hepatopancreas through blood in the form of phospholipids to the ovary (Li et al., 1994; Subramoniam, 2011). In the hepatopancreas, the contents of HUFA, SFA, and MUFA in stage Ⅴ of the FVP were significantly higher than those of the SVP. In addition, during the SVP, the contents of HUFA, SFA, and MUFA tended to increase from ovarian stage Ⅳ to Ⅴ; however, they decreased in the hepatopancreatic tissue during this period, which is inconsistent with the result of a previous study in *S. paramamosain* (Wu et al., 2020). This finding implies that *S. paramamosain* requires additional fatty acids stored in the hepatopancreas to be transported to the ovary to meet the nutritional requirement of secondary ovary maturation during SVP.

In the muscle, the contents of EPA and MUFA in stage Ⅴ of the SVP were significantly higher than those of the FVP. The contents of total protein, TAA, and EAA in the stage Ⅴ ovary during SVP were higher than those during FVP, which differed from the result of a previous study during FVP (Wu et al., 2020). As a previous study argued that the nutrients in the muscle were not involved in the supply of nutrients during ovarian development (Wu et al., 2020), the higher muscle nutrients during the SVP relative to those during the FVP may be due to the greater amount of nutrients that gradually accumulated in the muscle relative to that consumed, with the extension of the development time. Therefore, an increasing number of nutrients accumulate in the muscle.

### Analysis of the transport patterns of nutrients from the hepatopancreas to the ovary

**FIGURE 9:**
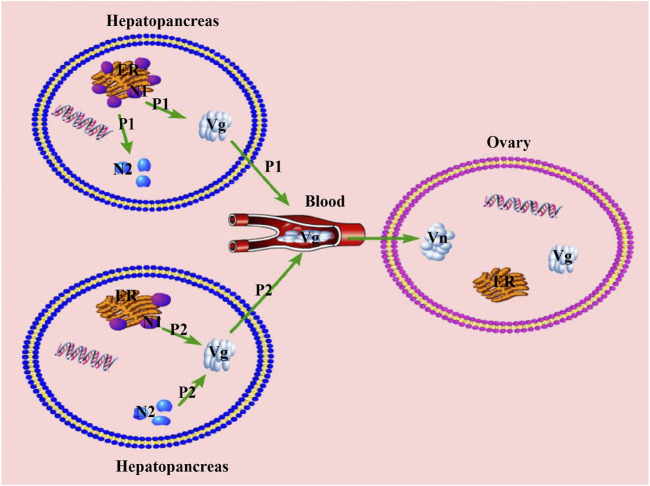
Two different patterns of nutritional transportation from hepatopancreas to the ovary during the vitellogenesis period. P1, patter one; P2, pattern two; ER, Endoplasmic reticulum; N1, Nutrient 1, synthesized by the endoplasmic reticulum; N2, Nutrient 2, stored in the hepatopancreas; Vg, Vitellogenin; Vn, Vitellin.

During the vitellogenesis period of crustaceans, *Sp-Vg* was mainly expressed in the hepatopancreas and ovary, where the hepatopancreas is the main tissue for the synthesis of Vg. The Vg in the hepatopancreas was processed, modified, and then transported through the hemolymph to the ovary to meet the requirements of ovarian maturation. In addition, some lipids and other nutrients that accumulated in the hepatopancreas needed to be transported to the ovary through the vehicles of Vg and Vn (Rodríguez-González et al., 2006; Subramoniam, 2011). Therefore, the differences in the vitellogenesis patterns may be mainly reflected in the differences in the transport of nutrients from the hepatopancreas. Based on previous reports, we can hypothesize that there are two different patterns of nutritional transportation from the hepatopancreas to the ovary (or vitellogenesis patterns) during the vitellogenesis period, as illustrated in Figure 9. In pattern one (P1), the capacity of nutrient transport (CNT) is relatively higher than that of their synthesis (the capacity of nutrients synthesis: CNS) in the hepatopancreas, and the nutrients are synthesized and then transported from hepatopancreas to the ovary. The excess nutrients are stored in hepatopancreas as internal storage of nutrients, revealing positive correlation or no significant correlation between the HSI and GSI; however, a gradual increase occurred during the ovarian maturation period, i.e., *S. paramamosain* (Wu et al., 2020) and *Nephrops norvegicus* (Rosa & Nunes, 2002). In pattern two (P2), the CNT is relatively lower than CNS in the hepatopancreas. In addition to transporting the nutrients synthesized by the hepatopancreas, the ovary needs to transport additional nutrients that are inherently stored in the hepatopancreas, indicating that the HSI and GSI are significantly negatively correlated during the ovarian maturation period (i.e., *E. sinensis* (Liu et al., 2011), *P. trituberculatus* (Wu et al., 2007), *Cherax quadricarinatus* (Rodríguez-González et al., 2006), *Litopenaeus vannamei* (Palacios et al., 2000), and *Macrobrachium rosenbergii* (Cavalli et al., 2001)). Based on the findings of this study, P1 may occur during the FVP of *S. paramamosain*, while P2 may occur during the SVP. The hypothetical answer for switching these two patterns is the inherent biological mechanism that mainly occurs in the hepatopancreas, which may be demonstrated by the decreasing capacity of nutritional synthesis in the hepatopancreas. For example, based on this study, as the hepatopancreas was also developing during the ovarian maturation of the FVP, both the GSI and HSI displayed an increasing trend during the FVP. However, during the SVP, due to the gradual degeneration of the hepatopancreas, the HSI gradually declined during the SVP, and *Sp-Vg* expression levels in the hepatopancreas and ovary during SVP were lower than those during the FVP. This is because the capacity of nutritional synthesis in the hepatopancreas during the SVP was lower than that during FVP. To complete the process of ovarian maturation, the nutrients required for ovarian maturation must be transported to the ovary. Therefore, numerous nutrients that are inherently stored in the hepatopancreas are preferentially transported to the ovary during a particularly short developmental period of the ovary (SVP), leading to a decrease in the nutritional value or further degeneration in the hepatopancreas. Of the two patterns, P2 may be supported by previous studies (Nan, 2005; Yu et al., 2007; Liu et al., 2011), illustrating a type of reproductive-priority behavior in which individuals prioritize reproductive needs when the nutrients required for growth conflict with those required for reproduction (Friggens, 2003). Therefore, to better adapt to the living environment and reproduce, P1 will be applied by the crab when the growth environment is more suitable; otherwise, P2 will be prioritized. For example, in some economically important crabs, the HSI decreased as GSI increased during overwintering or food scarcity based on previous studies (Haefner & Spaargaren, 1993; Mourente et al., 1994). In addition, P1 under a better growth environment (appropriate temperature and sufficient bait) would be better for breeding in aquaculture, and P2 may be used for commercial crab culture.

## Conclusion

This study examined the differences, including in GSI, HSI, histological morphology, and biochemical components, between FVP and SVP. Based on the results, the ovary of *S. paramamosain* can re-mature after spawning and can approximately attain the status of the first ovarian maturation, including the levels of nutrients and the diameter of oocytes. However, the hepatopancreas displayed a gradual deterioration trend during the SVP. In addition to providing a clearer understanding of the correlation between GSI and HSI, this study revealed most biochemical dynamics (in terms of fatty acids and amino acids) and the expression levels of *Sp-Vg* in the ovary and hepatopancreas. Further studies are needed to investigate the hepatopancreas dynamics of *S. paramamosain*, which is valuable for understanding the relationship between the nutritional supply of the ovary and hepatopancreas during ovarian development.

## Data Availability

The original contributions presented in the study are included in the article/Supplementary Material, further inquiries can be directed to the corresponding author.
